# Production of green diesel from catalytic deoxygenation of chicken fat oil over a series binary metal oxide-supported MWCNTs†

**DOI:** 10.1039/c9ra08409f

**Published:** 2020-01-02

**Authors:** N. Aliana-Nasharuddin, N. Asikin-Mijan, G. Abdulkareem-Alsultan, Mohd Izham Saiman, Fahad A. Alharthi, Abdulaziz Ali Alghamdi, Y. H. Taufiq-Yap

**Affiliations:** Catalysis Science and Technology Research Centre (PutraCAT), Faculty of Science, Universiti Putra Malaysia 43400 UPM Serdang Selangor Malaysia ckin_mijan@yahoo.com taufiq@upm.edu.my +603-89466758 +603-89466809; Department of Chemistry, Faculty of Science, Universiti Putra Malaysia 43400 UPM Serdang Selangor Malaysia; Chancellery Office, Universiti Malaysia Sabah 88400 Kota Kinabalu Sabah Malaysia; Institute of Advanced Technology, Universiti Putra Malaysia 43400 UPM Serdang Selangor Malaysia; Chemistry Department Science College, King Saud University PO Box 2455 Riyadh 11451 Saudi Arabia

## Abstract

Deoxygenation processes that exploit milder reaction conditions under H_2_-free atmospheres appear environmentally and economically effective for the production of green diesel. Herein, green diesel was produced by catalytic deoxygenation of chicken fat oil (CFO) over oxides of binary metal pairs (Ni–Mg, Ni–Mn, Ni–Cu, Ni–Ce) supported on multi-walled carbon nanotubes (MWCNTs). The presence of Mg and Mn with Ni afforded greater deoxygenation activity, with hydrocarbon yields of >75% and *n*-(C_15_ + C_17_) selectivity of >81%, indicating that decarboxylation/decarbonylation (deCOx) of CFO is favoured by the existence of high amount of lower strength strong acidic sites along with noticeable strongly basic sites. Based on a series of studies of different Mg and Mn dosages (5–20 wt%), the oxygen free-rich diesel-range hydrocarbons produced efficiently by Ni_10_–Mg_15_/MWCNT and Ni_10_–Mn_5_/MWCNT catalysts yielded >84% of hydrocarbons, with *n*-(C_15_ + C_17_) selectivity of >85%. The heating value of the green diesel obtained complied with the ultra-low sulphur diesel standard.

## Abstract 2

Deoxygenation processes that exploit milder reaction conditions under H_2_-free atmospheres appear environmentally and economically effective for the production of green diesel.

## Graphical abstract

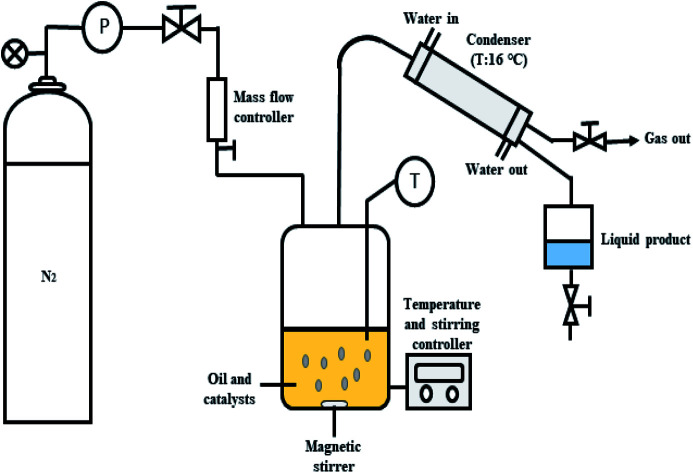

## 1. Introduction

Growing demand for fossil fuels, finite fossil fuel resources and related environmental issues have directed global attention towards the development of alternative fuels from renewable sources. Accordingly, many studies have been carried out to produce more efficient and inexpensive renewable fuels from various liquid biomass-derived feedstocks. Recently, biodiesel or fatty acid methyl esters (FAMEs) produced by transesterification processes from vegetable oils and animal fats have been commonly used as fuels in diesel engine transportation.^1^ Unfortunately, biodiesel is composed predominantly of highly oxygenated compounds that result in undesirable properties such as high viscosity, low oxidative stability, a high cloud point, high nitrogen oxide (NOx) emissions, and low energy density.^2^ In particular, oxygen-free hydrocarbon fuel known as green diesel is considered promising in this regard. Even though green diesel has a similar molecular structure to petroleum diesel but it has greater cetane number ranging from 85 to 99 (compared to 45 to 55 for petroleum diesel).^3^

Deoxygenation processes are particularly attractive for producing green diesel and the operational costs are relatively lower than the current upgrading process used in existing petroleum refineries, the hydrodeoxygenation (HDO) process. The hydrodeoxygenation process involves direct conversion of fatty acids *via* removal of oxygenated species, retaining the number of carbon atoms, with H_2_O as a by-product under H_2_ atmosphere.^4,5^ Meanwhile, deoxygenation processes involve the removal of oxygenated species in the forms of CO, CO_2_ and H_2_O *via* decarbonylation and decarboxylation (deCOx) under H_2_-free atmosphere.^6,7^ The hydrocarbon products formed typically contain one less carbon atom (C_*n*−1_) than the original fatty acid chain. The green diesel produced from deoxygenation exhibits better fuel characteristics – high heating value (HV), high cetane number (80–90), lower viscosity and high fuel stability; thus, it has been widely accepted by many research studies that green diesel is the most promising substitute for fossil-based diesel.^8^

Selecting the appropriate feedstocks for green diesel production is important for industrial practices. Usually, vegetable oil feedstock used for biofuel production consists of edible and non-edible oils. However, edible oils face problems related to the competition between food and fuel issues. Non-edible oils, such as *Jatropha* oil, rubber oil, *Ceiba* oil, karanja oil, *Sterculia* oil and castor oil are expensive, so they are unsuitable for biofuel production.^9^ Thus, the production of green diesel derived from animal fats has been widely explored. Commonly used animal fats include beef tallows, chicken fat, mutton fat and pork lard.^10–13^ Amongst them, chicken fat oil (CFO) offers a better alternative renewable source. Commonly, chicken is known as a staple food worldwide, and its production reaches about 107.1 million metric tons. ^14^ Fat contents in chicken poultry range from 10% to 12%.^15^ Thus, approximately 10.7–12.9 million metric tons of chicken fat is produced per year. CFO is composed of C16 and C18 fatty acids,^16^ so CFO is considered as ideal feedstock for diesel-range hydrocarbon (*n*-C_15_ and *n*-C_17_) production *via* deCOx pathways.

A study by Yang *et al.*^17^ discovered that solid catalysts such as noble metal, metal oxide and sulphided catalysts were highly efficient in promoting deoxygenation of fatty acid. Among those catalysts, noble metals such as Pd, Pt, and Rh were proven to be the most active metal promoters of deoxygenation activity and possessed greater affinity towards C–O bond cleavage *via* deCOx pathways.^18^ However, the high cost constraints made them unattractive. Metal sulphided catalysts such as NiMo and CoMo are commonly used in deoxygenation reactions. Unfortunately, sulphide-based catalysts cause sulphur leaching, which deactivates catalysts and contaminates products.^19^ As such, nonsulphur-based, inexpensive and highly active catalyst for deoxygenation should be developed. Interestingly, metal oxides are inexpensive and essentially free of sulphur. Metal oxides seem to be realistic deoxygenation catalysts for the future. Metal oxide catalysts, especially Ni-based, show activity comparable to noble metals in converting lipid-based feeds to liquid hydrocarbons. As discussed by Morgan *et al.*^20^ Ni on carbon support catalysts showed similar activity to that of Pd- and Pt-promoted catalysts at higher concentration, which suggested that Ni has good performance in replacing noble metals in deoxygenation. Oxides of other metals, such as Ce, W, Co, Fe, Cu, Mo, Zn, Mg and Ca, have also been used in deoxygenation under H_2_-free conditions.^21–24^ Interestingly, Ce and Cu offer better deoxygenation reaction selectivity in diesel-like hydrocarbon production. Aysu *et al.*^23^ studied the deoxygenation of jojoba oil over Ce-promoted catalysts and the results showed that the reaction occurred exclusively *via* deCOx, which yielded higher percentages of aliphatic compound. A similar case, with Cu-promoted catalysts studied by Loe *et al.*^24^ whereby the Cu-promoted catalyst was demonstrated to be active in removing the oxygen atoms from free-fatty-acid-derived oxygenates and yielded >90% of diesel-range hydrocarbons. Additionally, a basic promotion catalyst (MgO) also favoured the deCOx reaction. Tani *et al.*^22^ discovered that MgO-supported catalysts resulted in enhanced triglyceride cracking *via* decarboxylation, and the green diesel produced resembled conventional liquid fuel. Moreover, use of basic metal catalysts can suppress coke formation and offer greater catalyst stability.^25^ Parenthetically, no study has reported the use of Mn as catalyst in deoxygenation reactions. However, Mn was found to be active in the pyrolysis of sawdust and produced 48.5% of H_2_ gas.^26^

Instead of catalyst promoter, the catalyst support always plays a critical role in promoting the deoxygenation reaction. This is due to support being able to enhance active metal dispersion, simultaneously increasing the active sites for catalysis of the reaction.^27^ Carbon is a promising support, which can be attributed to the high specific area and the nature of carbon itself, being thermally stable, thereby minimising the sintering of the active metal during the deoxygenation reaction.^28^ Nanosized carbon supports, such as multi-walled carbon nanotubes (MWCNTs) have specific pore structures that offer better thermal stability than micron-size activated carbon supports, and the use of MWCNTs as catalyst support in the deoxygenation reaction has been recognised.^29^ As discussed by Asikin-Mijan *et al.*^30^ MWCNTs were used as catalyst supports in the deoxygenation of *Jatropha curcas* oil, resulting in high selectivity towards C_15_ + C_17_*via* the deCOx pathway and producing >80% hydrocarbon yield. However, the catalyst still favoured greater coke formation (4–5 wt%) than other carbon supported catalysts (∼2 wt%).^31^ Thus, in order to improve coke resistancy of MWCNT, hence this study highlighted the modification of MWCNTs through the incorporation of a series of binary metal oxide pairs (Ni–Mg, Ni–Mn, Ni–Cu, Ni–Ce) for CFO deoxygenation under H_2_-free conditions. The effect of metal concentration, within the range of 5–20 wt% on reaction activity was further investigated, and the green diesel produced was subjected to an HV test.

## 2. Experimental

### 2.1 Materials and methods

Multi-walled carbon nanotubes with purity > 95% were purchased from US Research Nanomaterials, Inc. Nickel(ii) nitrate hexahydrate (Ni(NO_3_)_2_·6H_2_O) with purity > 99%, magnesium(ii) nitrate hexahydrate (Mg(NO_3_)_2_·6H_2_O) with purity > 99%, and manganese(ii) acetate tetrahydrate (C_4_H_6_MnO_4_·4H_2_O) with purity > 98% were obtained from R&M Company. Copper(ii) nitrate trihydrate (Cu(NO_3_)_2_·3H_2_O) with purity > 99% and cerium(iii) nitrate hexahydrate (CeH_12_N_3_O_15_) with purity > 99% were procured from Merck and Acros, respectively. Phosphoric acid (H_3_PO_4_) with purity > 85% was purchased from J.T Baker. The liquid products for standard gas chromatography (GC) analysis, C_8_–C_20_ alkene and alkane standard solutions and the internal standard 1-bromohexane were purchased from Sigma Aldrich and used without further purification. For dilution, *n*-hexane (GC grade) with purity > 98% from Merck was used. The feedstock involved was CFO obtained from raw chicken fat waste from a local Serdang market in Malaysia. The chicken fat waste was heated at 120 °C for 3 h and filtered to remove any solid residue. The resultant samples are referred to as CFO. The CFO was further treated at 120 °C in an oven to ensure the water content was <0.5 wt%. Water content was measured by using the ASTM E203-08 method. Table 1 shows that the CFO was composed of triglycerides (TGs, 83%), free fatty acids (FFAs, 17%) with a moisture content of 0.3 wt%. According to the analysis, the fatty acid composition of CFO was mainly saturated C_16_ (palmitic) and unsaturated C_18_ (oleic) fatty acids.

**Table 1:** Properties of CFO

Properties	Value
Moisture content (wt%)	0.30
Acid value (mg KOH per g)	33.66
FFA value (%)	16.83
Fatty acid composition of oil (%)	
Palmitic acid (C16:0)	29.56
Stearic acid (C18:0)	9.45
Oleic acid (C18:1)	57.61
Linoleic acid (C18:2)	3.38

### 2.2 Catalyst synthesis

All catalysts were synthesised *via* a wet-impregnation method. Initially, 6 g of MWCNT support was chemically activated with 90 mL of phosphoric acid (H_3_PO_4_) with reflux heating at a temperature of 150 °C for 24 h. Then, the H_3_PO_4_ was removed using hot distilled water until the solution reached pH 7. The activated MWCNTs were then dried in an oven at 120 °C. The activated MWCNTs were then impregnated with 10 wt% of Ni(NO_3_)_2_·6H_2_O and 10 wt% of C_4_H_6_MnO_4_·4H_2_O under continuous stirring for 6 h at ambient temperature. The solid was dried in an oven at 120 °C and further calcined at 550 °C for 4 h under inert conditions and the catalyst denoted as Ni_10_–Mn_10_/MWCNT. The procedures above were repeated, replacing the Mn salt with Mg(NO_3_)_2_·6H_2_O, CeH_12_N_3_O_15_ and Cu(NO_3_)_2_·3H_2_O. The catalysts were denoted Ni_10_–Mg_10_/MWCNT, Ni_10_–Ce_10_/MWCNT and Ni_10_–Cu_10_/MWCNT, respectively. Furthermore, Ni_10_/MWCNT was prepared *via* a similar preparation route. In addition, the optimisation of metal dosage of the most effective metals (Mg, Mn) was performed by varying the metal content from 5 to 20 wt% with Ni remaining constant at 10 wt%. The catalysts were denoted Ni_10_–Mg_*n*_/MWCNT and Ni_10_–Mn_*n*_/MWCNT, where *n* = 5, 10, 15 and 20.

### 2.3 Catalyst characterisation

Powder X-ray diffraction (XRD) analysis was performed to determine the dispersion state and chemical composition of the modified MWCNT catalysts before and after reaction. The XRD analysis was carried out using Shimadzu diffractometer, model XRD-6000. The acidity and basicity of all catalysts were measured using temperature programmed desorption with NH_3_ and CO_2_ as the probe molecule (TPD-NH_3_ and TPD-CO_2_). The analysis was carried out using a Thermo Finnigan TPD/R/O 1100 instrument equipped with a thermal conductivity detector (TCD). The catalyst (∼0.05 g) was pre-treated in N_2_ gas flow for 30 min at 250 °C and then with NH_3_ gas for 1 h at ambient temperature to allow adsorption of NH_3_ onto the surfaces. Then, the excess NH_3_ was flushed with N_2_ gas flow at 20 mL min^−1^. The desorption of NH_3_ from the acid sites of the catalyst was detected by TCD under He gas flow (30 mL min^−1^) from 50 to 900 °C and held for 30 min. The adsorption and desorption of CO_2_ was carried out in a similar way to the TPD-NH_3_ method. The field emission scanning electron microscopy-energy dispersive X-ray (FESEM-EDX) analysis was used to investigate the morphology and to determine the elemental composition of the catalysts. The FESEM images were recorded on a LEO 1455 VP electron microscope. The FESEM connected to EDX used a Rayny EDX-720 spectrometer for determination of the elemental composition of C, O, Ni, Mg, Mn, Cu and Ce. The TGA instrument (TGA 1000i, Instrument Specialists Inc, USA) was used to determine the extent of coke/carbon deposition on the spent catalyst under atmospheric conditions. The powder samples were heated in the range of 25 to 1000 °C at a heating rate of 10 °C min^−1^ under 40 mL min^−1^ air flow.

### 2.4 Catalytic deoxygenation of CFO

**Fig. 1:**
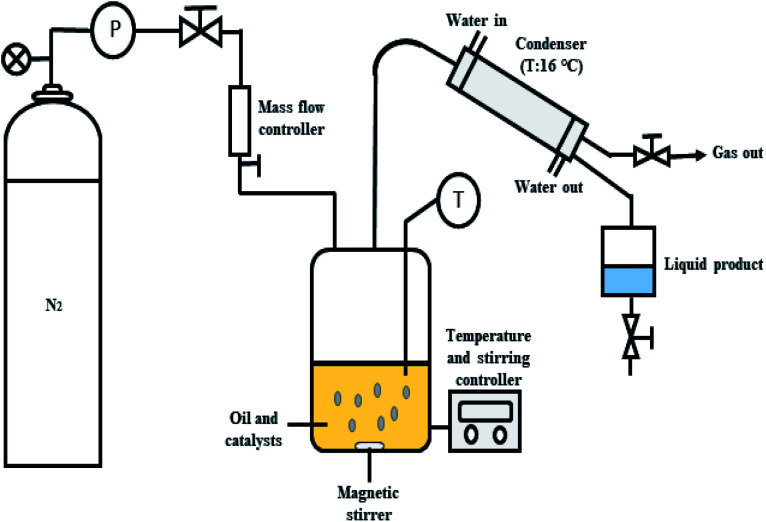
Schematic diagram for the semi-batch reactor in deoxygenation of CFO.

The deoxygenation of CFO was performed in a mechanically stirred 250 mL semi-batch reactor, as shown in Fig. 1. Initially, 10 g of CFO and 3 wt% of catalyst were added into the reactor. Before each reaction, the oxygen in the reactor produced by the heating process was removed by purging with N_2_ gas at flow rate of 20 mL min^−1^ with constant stirring of the reaction mixture. The temperature was increased to the desired temperature of 350 °C and the reaction maintained for 2 h under inert gas flow (N_2_, 50 mL min^−1^). The condensable liquid product was condensed using an external water-cooled circulator into a collection vessel. The liquid products were further weighed and analysed using total acid number (TAN) test, gas chromatography with flame ionisation detector (GC-FID) and gas chromatography-mass spectrometry (GC-MS). The gaseous products were collected in a gas bag 1 h before the reaction finished and the gases further analysed by GC-TCD. All the reactions were repeated three times and the results evaluated as the average of the three repetitions. Mass-balance analyses were conducted for all deoxygenation reactions. The solid catalysts were separated by mixing the liquid residue inside the reactor with hexane to determine the mass of the retained products (char and residue) after reaction. The hexane was removed *via* rotary evaporation and the dark viscous liquid identified as char + residue.

### 2.5 Product analysis

The deoxygenated liquid products were identified using C_8_–C_20_ alkane and alkene standards. The liquid products were analysed quantitatively using GC (Agilent GC-14B equipped with an HP-5 capillary column, length 30 m × inner diameter 0.32 mm × film thickness 0.25 μm) operating at 300 °C. The liquid product was diluted with GC grade *n*-hexane prior to the yield analysis. 1-Bromohexane was used as internal standard. A 2 μL aliquot was injected into the GC column at an inlet temperature 250 °C and helium gas served as the carrier gas. The initial temperature of the oven was set at 40 °C and held for 6 min. Then, the oven temperature was ramped to 300 °C at a heating rate of 7 °C min^−1^. The TAN of the CFO and the liquid products were identified by following the standard procedure of AOAS Cd 3 d-63, which is referred to as the classical titration method eqn (1):1
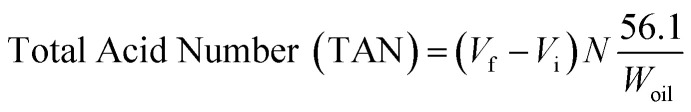


The determination of hydrocarbon yield (*X*) on the catalyst performance were evaluated by GC-FID using eqn (2).^30^2
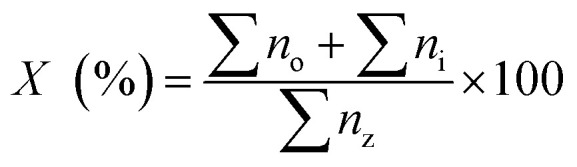
where *n*_o_ = peak area of alkenes (C_8_–C_20_), *n*_i_ = peak area of alkanes, *n*_z_ = peak area of the product. The hydrocarbon selectivity (*S*) of the deoxygenated products was determined from eqn (3):3
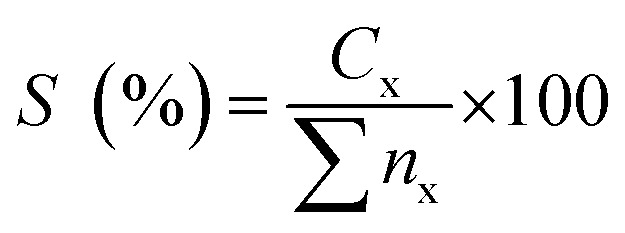
where *C*_x_ = peak area of desired hydrocarbon fraction and *n*_x_ = peak area of hydrocarbons.

Product distribution of CFO and deoxygenated liquid products were characterised qualitatively using GC-MS (Shimadzu model QP5050A) equipped with a non-polar DB-5HT column (30 m × 0.25 mm × I.D. μm) with splitless inlet. The samples were diluted with GC grade *n*-hexane of purity > 98%. The fraction peaks from the GC-MS spectra were identified using the National Institute of Standards and Testing (NIST) library. The major products identified by GC-MS (hydrocarbon fractions, carboxylic acids, alcohols, cyclic compounds and ketones) were compared with the probability match between 95 and 100%. The Fourier transform infrared spectrometry (FTIR) analysis was performed using a Nicolet 6700 spectrometer from Thermo Scientific. This analysis was used to identify the functional groups present in CFO and the liquid products. The spectrometer used a resolution of 4 cm^−1^ within the range 300–4000 cm^−1^. The gaseous products were analysed using a Shimadzu GC-8 A GC-TCD *via* an offline method with stainless steel adsorption column packed with molecular sieve. The higher HV of the liquid products were measured in a bomb calorimeter according to the ASTM D2015 standard method.

## 3. Results and discussion

### 3.1 Characterisation of the catalysts

**Fig. 2:**
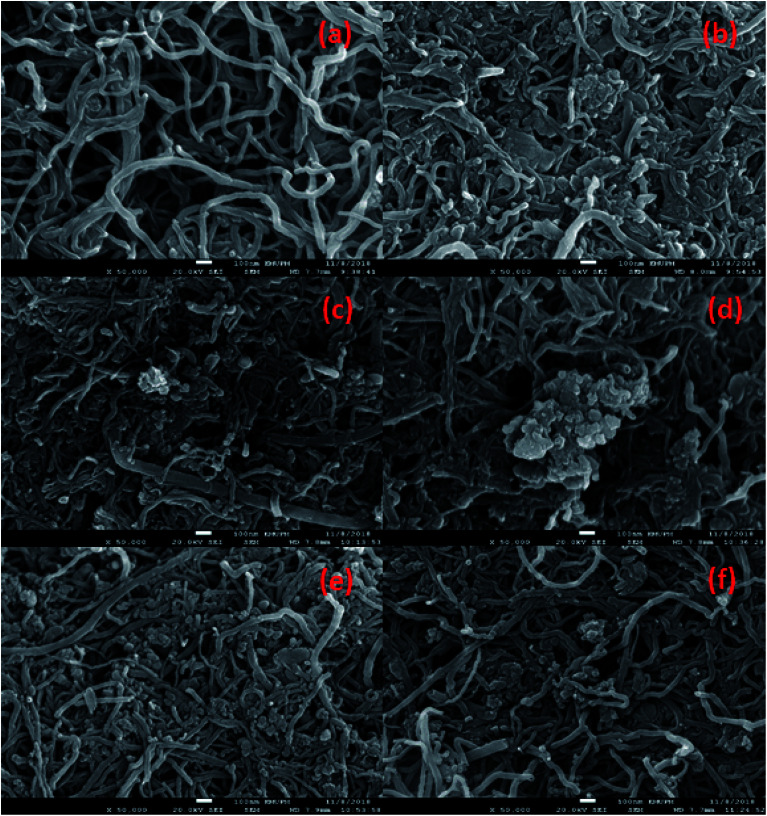
FESEM-EDX analysis for (a) MWCNT, (b) Ni_10_/MWCNT, (c) Ni_10_–Mg_10_/MWCNT, (d) Ni_10_–Mn_10_/MWCNT, (e) Ni_10_–Ce_10_/MWCNT and (f)Ni_10_–Cu_10_/MWCNT.

The morphological structure and the elemental composition of the MWCNTs and MWCNT-supported metal oxide catalysts are shown in Fig. 2a–f and Table 2. The FESEM images showed that the MWCNTs exhibited a nanotube structure with diameter < 100 nm; meanwhile, the obtained MWCNT-supported metal catalysts were found to form compact aggregates, which might due to the dopant effect resulting in aggregation (Fig. 2b–f). This is consistent with previous findings; dopant species were reported assembled into aggregates on the MWCNTs when synthesised by a wet-impregnation method.^32^ It is noteworthy to observe that the nanotube structure of MWCNTs remained unchanged after introduction of the metal species. Notably, the NiO in Ni_10_/MWCNT displayed bulky dense aggregates (Fig. 2b); however, with further introduction of Mn, Cu, Ce and Mg the dense aggregates were no longer obvious (Fig. 2c–f). It is reasonable to state that the homogeneous morphology and the small size of metal particles on the MWCNT surface could be achieved by the introduction of the binary metal oxide system. The elemental composition showed that the carbon content was the highest (>75%) followed by oxygen (<19%). Thus, it is expected that the carbon-rich catalysts could act as effective barriers to sintering, providing excellent mechanical properties for the deoxygenation reaction, thereby improving catalyst stability.^33^ The high content of oxygen corresponds to the formation of the oxide phase. Overall, the metal species (Mg, Cu, Mn, Mg, Ni) were found in trace amounts (1.7–7.7%) in which the percentages were lower than the theoretical, which might due to the metal species being embedded within the MWCNTs and not fully dispersed on the MWCNT surface.^34^

**Table 2:** Elemental composition of the MWCNT-supported catalysts

Catalysts	Elemental composition (%)						
	C	O	Ni	Mg	Mn	Ce	Cu
MWCNT	95.4	4.6	—	—	—	—	—
Ni_10_/MWCNT	91.14	7.19	1.67	—	—	—	—
Ni_10_–Mg_10_/MWCNT	75.26	19.73	2.64	2.37	—	—	—
Ni_10_–Mn_10_/MWCNT	84.90	9.53	2.56	—	3.01	—	—
Ni_10_–Ce_10_/MWCNT	83.90	11.66	2.65	—	—	1.79	—
Ni_10_–Cu_10_/MWCNT	79.84	9.39	7.72	—	—	—	3.04

**Fig. 3:**
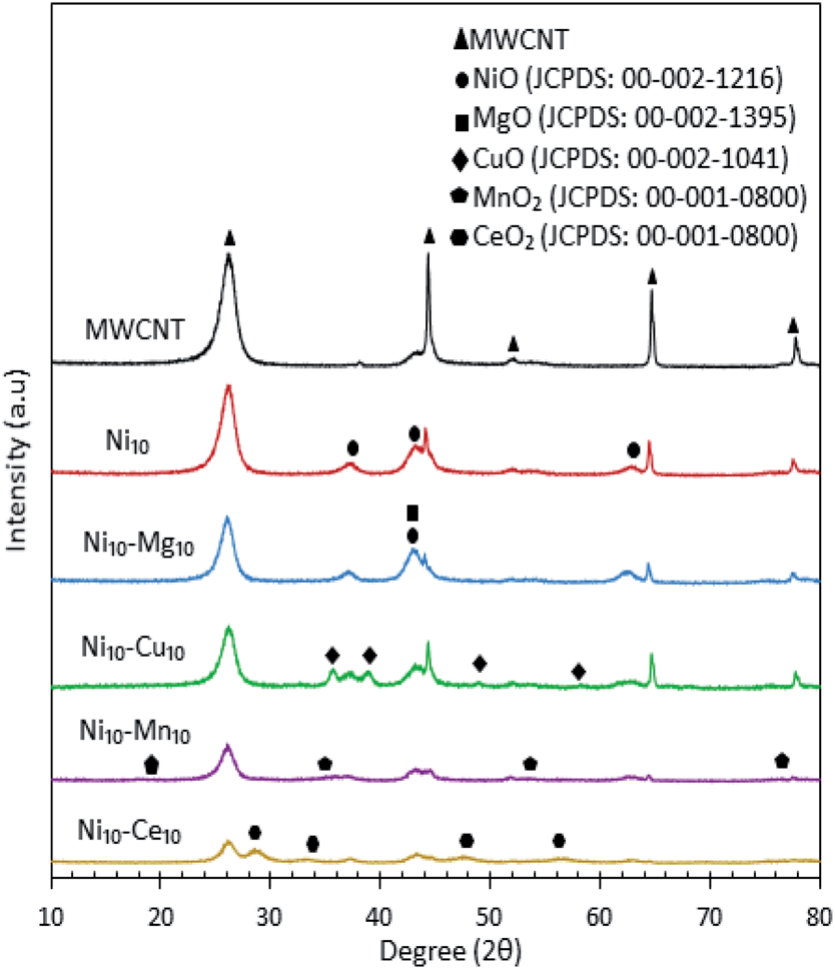
X-ray diffraction patterns for the MWCNT and MWCNT supported catalysts.

The XRD patterns for all catalysts are shown in Fig. 3, which showed that the MWCNT exhibits diffraction peaks at 2*θ*: 26.22°, 44.31°, 52.18°, 64.67° and 77.74°, which are assigned to the MWCNTs planes of (002), (100), (110), (004) and (006), respectively.^30^ Based on the XRD analysis, all the metal species were present in separate oxide phases. All Ni-containing catalysts exhibited XRD peaks corresponding to the cubic structure of NiO at 2*θ*: 37.05°, 43.14° and 62.89° (JCPDS file no.: 00-002-1216). The Ni_10_–Mg_10_/MWCNT catalysts exhibited a diffraction peak at 2*θ*: 42.92° (JCPDS file no.: 00-002-1395), which is assigned to the hexagonal structure of MgO. The XRD diffraction of Ni_10_–Cu_10_/MWCNT exhibited the monoclinic structure of CuO phase at 2*θ*: 35.71°, 38.98°, 48.93° and 58.17° (JCPDS file no.: 00-002-1041). In the case of Ni_10_–Mn_10_/MWCNT, the cubic structure of MnO was evident from the peaks at 2*θ*: 18.16°, 35.13°, 53.52° and 76.17° (JCPDS file no.: 00-001-0800). Furthermore, Ni_10_–Ce_10_/MWCNT displayed XRD diffraction peaks at 2*θ*: 28.74°, 33.27°, 47.55° and 56.36° (JCPDS file no.: 00-001-0800), corresponding to the cubic structure of CeO_2_. The crystalline peaks for MWCNT reduced remarkably after incorporation with the active metal species, attributed to the intercalation of metal oxides on the MWCNTs and thus promoting a higher dispersion of MWCNTs. The mean crystallite size of catalyst was determined by the Debye–Scherrer equation based on the highest intense peak centred at 2*θ*: 26.22°, and the results are tabulated in Table 3. The crystallite sizes follow the order Ni_10_–Ce_10_/MWCNT < Ni_10_–Mg_10_/MWCNT < Ni_10_/MWCNT < MWCNT < Ni_10_–Cu_10_/MWCNT < Ni_10_–Mn_10_/MWCNT. The crystallite size of Ni_10_–Ce_10_/MWCNT was the smallest (∼18 nm), suggesting a strong interaction between Ni and Ce species enhancing the MWCNT dispersion.^35^

**Table 3:** Textural and physicochemical properties of the MWCNT-supported catalysts

Catalysts	XRD^a^	TPD-NH_3_^b^		TPD-CO_2_^c^	
	Crystallite size^a^ (nm)	Temperature (°C)	Amount of NH_3_ absorbed (μmol g^−1^)	Temperature (°C)	Amount of CO_2_ absorbed (μmol g^−1^)
MWCNT	64.99	—	—	—	—
Ni_10_/MWCNT	37.89	512	1822	506	2263
Ni_10_–Mg_10_/MWCNT	28.47	337, 620	775, 2026	337, 637	421, 782
Ni_10_–Mn_10_/MWCNT	75.83	535	2590	540	904
Ni_10_–Ce_10_/MWCNT	18.98	492	2090	491	804
Ni_10_–Cu_10_/MWCNT	75.75	643	2242	632	700

a Measured by using Scherrer equation from XRD data.

b Determined by TPD-NH_3_ analysis.

c Determined by TPD-CO_2_ analysis.

Fig. S1† shows the TGA profile for the thermal behaviour of MWCNTs and MWCNT-supported metal oxide catalysts. The MWCNT support was thermally stable up to 500 °C, while Ni_10_–Mn_10_/MWCNT, Ni_10_–Cu_10_/MWCNT, Ni_10_–Mg_10_/MWCNT and Ni_10_–Ce_10_/MWCNT showed weight loss changes at 400 °C. The weight loss within the temperature the range 400–650 °C is typically due to the oxidation of MWCNTs.^36^ The reduction in thermal stability of MWCNTs by incorporation of binary metal oxide promoters is due to the presence of structural imperfections (vacancies and dislocations).^37^ The Ni_10_–Mg_10_/MWCNT showed additional weight losses at a lower temperature (<300 °C), which is attributed to the vaporisation of physically adsorbed water.^38^ Although the Ni_10_–Mg_10_/MWCNT had been thermally calcined at 500 °C, the water weight loss stage still remained, suggesting that MgO is naturally hygroscopic in nature and tends to absorb moisture forming Mg(OH)_2_.^39^ Overall, Ni_10_–Mn_10_/MWCNT, Ni_10_–Cu_10_/MWCNT and Ni_10_–Ce_10_/MWCNT showed higher stability (decomposing at temperatures >400 °C), leading to a greater reaction stability during deoxygenation of CFO at 350 °C.

Acid sites typically provide active sites for promoting C–C bond cleavage *via* cracking reactions; however, catalysts with excessively acidic sites would initiate coke formation during deoxygenation and deactivation of the catalyst. Interestingly, incorporation of basic sites with acid sites have been found to be successful in suppressing coke formation, while simultaneously retaining deoxygenation activity. Olusola *et al.*^40^ reported that the presence of basic sites is necessary for enhancing C–O bond cleavage through decarboxylation. In this regard, it suggests that the acidic and basic sites play major roles in enhancing the deoxygenation reaction. Fig. S2a† and Table 3 show the acidity of the synthesised MWCNTs and MWCNT-supported metal oxide catalysts. Based on the TPD-NH_3_ profile obtained, no acidity was detected on the MWCNTs, which likely corresponds to the amphoteric properties of carbon in MWCNTs.^30^ Nevertheless, large desorption peaks were observed for MWCNT-supported metal oxide catalysts at temperatures within the range 300–910 °C, indicating the presence of medium (300–500 °C) and strong acid sites (>500 °C). The total acid densities are ranked in the increasing order MWCNT < Ni_10_/MWCNT < Ni_10_–Ce_10_/MWCNT < Ni_10_–Cu_10_/MWCNT < Ni_10_–Mn_10_/MWCNT < Ni_10_–Mg_10_/MWCNT. The high acidity produced by MWCNT-supported metal oxide catalysts affirmed the role of metal species in enhancing the acidic sites of MWCNTs. This also in agreement with Wan *et al.*^41^ who proposed that the acidity of the acid sites is increased remarkably with the addition of active metal species. The basicity profile and its strength were analysed using TPD-CO_2_, and the results are displayed in Fig. S2b† and Table 3. All catalysts exhibited similar desorption peaks at temperatures of 100–500 °C and >500 °C, which indicates the presence of weak, medium and strong basic sites. The trend of the basicity density is arranged as follows: MWCNT < Ni_10_–Cu_10_/MWCNT < Ni_10_–Ce_10_/MWCNT < Ni_10_–Mn_10_/MWCNT < Ni_10_–Mg_10_/MWCNT < Ni_10_/MWCNT. It is worthy of mention that all MWCNT-supported metal oxide catalysts exhibited strong basic sites with the exception of Ni_10_–Ce_10_/MWCNT. This finding was in agreement with Wang *et al.*^42^ who suggested that addition of Ce species results in minor effects in changing the basic sites. Based on the TPD-NH_3_ and TPD-CO_2_ findings, Ni_10_–Cu_10_/MWCNT showed highest strength of acid and basic sites, respectively. This can be attributed to the synergistic effect of the acid–base interaction between CuO and NiO on the MWCNT surface.^30^

### 3.2 Catalytic deoxygenation of CFO

**Fig. 4:**
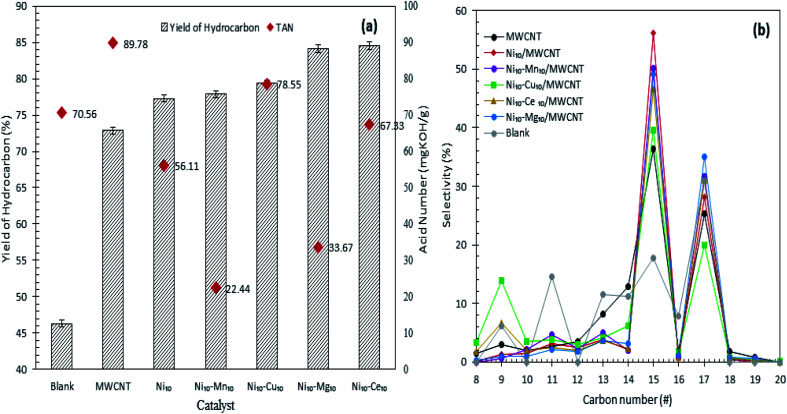
(a) Yield of hydrocarbon and (b) product selectivity of deoxygenized liquid product from catalysed deoxygenation. Reaction condition: *T* = 350 °C, 2 h reaction time, 3 wt% of catalyst loading.

Fig. 4a and b shows the hydrocarbon yield from catalytic deoxygenation of CFO over MWCNTs and MWCNT-supported metal oxide catalysts at 350 °C for 2 h reaction time using 3 wt% catalyst loading under N_2_ flow. As depicted in Fig. 4a, the blank reaction showed the lowest deoxygenation activity, confirming the occurrence of a catalytic reaction. MWCNT-supported metal oxides afforded greater hydrocarbon *n*-(C_8_–C_20_) yield than did MWCNTs in the deoxygenation reaction. This confirmed the necessity of the presence of the active metal in improving the deoxygenation activity for production of fuel-like hydrocarbons. The yield of hydrocarbons increased in the order blank < MWCNT < Ni_10_/MWCNT < Ni_10_–Mn_10_/MWCNT < Ni_10_–Cu_10_/MWCNT < Ni_10_–Mg_10_/MWCNT < Ni_10_–Ce_10_/MWCNT. Maximum hydrocarbon yield (∼84%) was observed over Ni_10_–Ce_10_/MWCNT, suggesting that the presence of cerium oxide contributed great catalytic activity due to the high dispersion of MWCNTs.^43^ Obviously, binary metal-oxide-promoted catalysts rendered higher formation of *n*-(C_8_–C_20_) than did Ni_10_/MWCNT, which is attributable to the remarkable increase in acidic sites (Table 3).^44^ CFO is mainly composed of C_16_ (palmitic) and C_18_ (stearic, oleic and linoleic) fatty acids (Table 1). Thus, deoxygenation of CFO *via* removal of oxygenated species (CO_2_, CO) *via* the deCOx reaction will lead to the formation of C_15_ and C_17_ hydrocarbon fractions.^45,46^ As demonstrated in Fig. 4b, *n*-C_15_ and *n*-C_17_ were obtained as the main hydrocarbon products from catalysed CFO deoxygenation. The *n*-(C_15_ + C_17_) selectivity increased in the order blank < MWCNT ∼ Ni_10_–Cu_10_/MWCNT < Ni_10_–Ce_10_/MWCNT < Ni_10_–Mn_10_/MWCNT < Ni_10_–Mg_10_/MWCNT < Ni_10_/MWCNT. As expected, the blank reaction resulted in lower *n*-(C_15_ + C_17_) selectivity (48%) and higher light-hydrocarbon-fraction *n*-(C_9_–C_14_) formation (∼42%). By contrast, metal oxide-supported MWCNT selectively deoxygenised the CFO to *n*-(C_15_ + C_17_). This finding suggested that C–O bond cleavage occurred *via* a deCOx reaction.

Interestingly, Ni_10_/MWCNT is more favourable towards deCOx reaction and produced the highest *n*-(C_15_ + C_17_) (84%) but lower hydrocarbon yield (77%), implying that the deCOx reaction is favoured by basic-site-rich catalysts (2263 μmol g^−1^). The low hydrocarbon yield indicated that NiO plays a critical role in increasing C–C bond cleavage *via* cracking and increasing the amount of volatile product, so proving that Ni_10_/MWCNT facilitated simultaneous cracking-deCOx reactions. It can be seen that Ni_10_–Mg_10_/MWCNT and Ni_10_–Mn_10_/MWCNT were also effective in the deCOx reaction, rendering high selectivity towards *n*-(C_15_ + C_17_) (81–83%). Similarly, these catalysts were also effective in converting the acidic compounds to non-acidic compounds, showing the lowest TAN value of 22–33 mg KOH per g (Fig. 4a) and outperformed Ni_10_–Ce_10_/MWCNT (TAN = 67 mg KOH per g, 77% *n*-(C_15_ + C_17_) selectivity). Indeed, the efficiency of the Ni_10_–Mg_10_/MWCNT and Ni_10_–Mn_10_/MWCNT catalysts in the deCOx reaction can be explained in terms of high basic (*T*_max_ = 540–637 °C) and acid (*T*_max_ = 535–620 °C) strengths.^33,47^ Similarly, high acidic and basic strengths were observed for Ni_10_–Cu_10_/MWCNT but the *n*-(C_15_ + C_17_) was lower (59%) and the percentage of light hydrocarbon fractions (C_8_, C_9_, C_10_, C_11_–C_14_) was highest. This can be inferred from the existence of strong acidic sites (*T*_max_ = 643 °C) simultaneously rendering greater occurrence of C–C bond cleavage.^45^ Although CFO has higher C_18_ fatty acid content (>50%) the liquid product is rich in *n*-C_15_ hydrocarbons (deoxygenated C_16_ fatty acids), which is implies by the cracking of deoxygenation products or C_18_ fatty acids.^31^

**Fig. 5:**
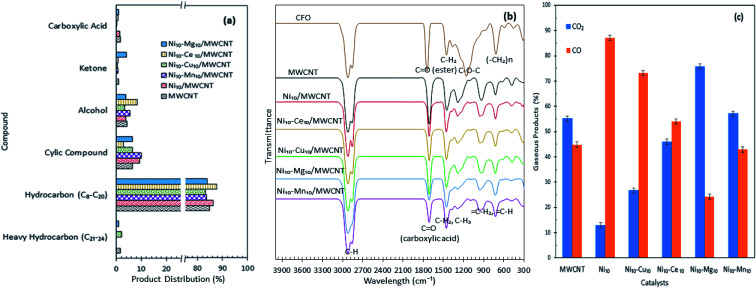
(a) Product distribution, (b) FTIR spectra of the CFO and deoxygenated liquid product and (c) composition of the gaseous products obtained from the catalysed deoxygenation. Reaction condition: *T* = 350 °C, 2 h reaction time, 3 wt% of catalyst loading.

Product distribution of the deoxygenated liquid product are displayed in Fig. 5a. Based on the GC-MS result, all the catalysts showed higher *n*-(C_8_–C_20_) hydrocarbon distribution (>85%) and with almost identical hydrocarbon percentages, suggesting that MWCNT-supported metal oxide catalysts are highly promising for promoting the deoxygenation activity and converting the CFO to hydrocarbon-like structures. The Ni_10_–Ce_10_/MWCNT catalyst showed the highest *n*-(C_8_–C_20_) hydrocarbon distribution (88%), which is in line with the GC-FID result. Furthermore, the deoxygenated liquid product also showed the presence of non-oxygenated compounds (heavy C > 20 and cyclic hydrocarbons) (1–10%) and oxygenated intermediate compounds (ketones, alcohols, carboxylic acids) (0.4–8%) (Table S1†). Notably, cyclic compounds and alcohols were pronounced in all liquid products (3–10%). The result also revealed that Ni_10_–Mg_10_/MWCNT favoured ketonisation side reactions and yielded the highest formation of ketone compounds (4%). This corresponds to the basicity of MgO itself, which prompted the occurrence of ketonisation forming coupling products.^48^ The CFO and deoxygenated liquid products were further analysed by FTIR. The results are displayed in Fig. 5b. The FTIR spectra of CFO showed the main absorption bands at 2917 cm^−1^ and 2850 cm^−1^ (–CH stretching), 1741 cm^−1^ (–C=O ester stretching), 1455 cm^−1^ (–CH_2_ bending), 1148 cm^−1^ (C–O–C stretching) and 703 cm^−1^ (–(CH)_*n*_– bending). The liquid product rendered primarily alkane and alkene functional groups with absorption peaks at 2911 cm^−1^ and 2827 cm^−1^ (–CH stretching), 1458 cm^−1^ (–CH_2_ bending), 1281 cm^−1^ (–CH_3_ bending), 922 cm^−1^ (=CH_2_ bending) and 719 cm^−1^ (–(CH)_*n*_− bending). It was notable that there was a significant shifting of the C=O stretching band from 1741 cm^−1^ (ester) in CFO to 1660 cm^−1^ (carboxylic acid) in the liquid products, indicating the formation of acid intermediates from esters *via* triglyceride cracking. In addition, the reduction in intensities of the C=O peak were used to determine the efficiency of deoxygenation. The reductions in the peak intensities were comparable with the reduction in oxygen content in the product.^49^ By comparing the deoxygenised liquid product, Ni_10_–Mg_10_/MWCNT and Ni_10_–Mn_10_/MWCNT showed significant decreases in C=O intensity, suggesting higher deoxygenation activity. The absence of the C–O–C band at 1148 cm^−1^ indicated that a triglyceride ester bond was eliminated possibly to produce free fatty acids.

The gaseous products of the reaction were further analysed through GC-TCD (Fig. 5c), considering that the deCOx reaction favoured the removal of C–O-containing species in the forms of CO, CO_2_ and H_2_O. The results revealed that the gaseous products were composed mainly of CO_2_ and CO, which confirmed the removal of C–O-containing species *via* decarboxylation and decarbonylation pathways. Aside from these pathways, side reactions, such as water gas shift (WGS) and Boudouard reactions, may occur. WGS reaction is reversible, that is, CO and H_2_O are converted into CO_2_ and H_2_ as expressed in eqn (4), while Boudouard reaction is a reaction between C and CO_2_ to produce CO as presented in eqn (5):

4 CO_(g)_ + H_2_O_(g)_ ⇌ CO_2(g)_ + H_2(g)_,

5 CO_2_ + C ⇌ 2CO.

Ni_10_/MWCNT exhibited the highest CO gaseous (87%), indicating that decarbonylation is the predominantly involved route.^50^ Ni edges pose greater C affinity and are susceptible to the Boudouard reaction.^51^ Thus, a catalyst containing Ni may partially undergo a Boudouard reaction to form CO. Notably, the gaseous product from Ni_10_–Mg_10_/MWCNT-catalysed deoxygenation contained the highest amount of CO_2_ species. This result suggested that numerous basic sites present in the Ni_10_–Mg_10_/MWCNT catalyst can lead to CO_2_ formation *via* decarboxylation.^22^ In Table 3 (TPD-CO_2_), the trend of the basic sites exhibited the following order: Ni_10_–Mg_10_/MWCNT > Ni_10_–Mn_10_/MWCNT > Ni_10_–Ce_10_/MWCNT > Ni_10_–Cu_10_/MWCNT. For instance, the CO_2_ production followed the same trend. This finding implies that more basic sites promote decarboxylation, thus producing high amounts of CO_2_.

### 3.3 Effect of Mg and Mn dosage on deoxygenation of CFO

Based on the screening of MWCNT-supported metal oxide catalysts in the deoxygenation reaction, it was found that incorporation of Mg and Mn on Ni/MWCNTs resulted in higher deoxygenation activity with product selectivity towards *n*-(C_15_ + C_17_) hydrocarbons. Hence, the effects of Mg and Mn promotion on CFO deoxygenation were further studied by varying the metal dosage within the range of 5 to 20 wt%. Based on XRD analysis, the Ni_10_–Mg_*n*_/MWCNT and Ni_10_–Mg_*n*_/MWCNT (*n* = 5, 10, 15, 20) exhibited the presence of cubic NiO, hexagonal MgO, and cubic MnO structures (Fig. S3†). The peak intensity for MWCNTs decreased with the increased of dosage of MgO and MnO species, which is due to the intercalation of metal oxides on the MWCNTs, hence promoting higher dispersion of MWCNTs. In worthy of mention that the NiO peak intensified with the incorporation of a high dosage of Mg and reduced with the addition of Mn-rich species, indicating that at high Mn loading, the NiO was present in small particles. NiO agglomeration took place at bulk at high Mg loading.^52^ As expected, the crystallite size of NiO determined at 2*θ*: 62.89° in Ni–Mn/MWCNT revealed a decrease in size from 17 nm to 10 nm with the increase in Mn dosage (Table 4). Meanwhile, NiO crystallite size increased to >78% with the addition of Mg species within the range of 5 to 20 wt%.

**Table 4:** Textural and physicochemical properties of the MWCNT-supported catalysts

Catalysts	XRD^a^	TPD-NH_3_^b^		TPD-CO_2_^c^	
		Acid sites (μmol g^−1^)		Basic sites (μmol g^−1^)	
	Crystallite size^a^ of NiO (nm)	Weak + medium, 50–500 °C	Strong, >500 °C	Weak + medium, 50–500 °C	Strong, >500 °C
Ni_10_–Mg_5_/MWCNT	13.68	—	3013	473	1387
Ni_10_–Mg_10_/MWCNT	15.38	775	2026	421	782
Ni_10_–Mg_15_/MWCNT	17.56	436	2142	957	1205
Ni_10_–Mg_20_/MWCNT	17.63	1815	2909	1199	876
Ni_10_–Mn_5_/MWCNT	17.59	—	3121	242	2683
Ni_10_–Mn_10_/MWCNT	12.32	—	2590	—	904
Ni_10_–Mn_15_/MWCNT	10.28	—	1845	—	1170
Ni_10_–Mn_20_/MWCNT	10.27	—	515	—	1590

a Measured by using Scherrer equation from XRD data of NiO peak.

b Determined by TPD-NH_3_ analysis.

c Determined by TPD-CO_2_ analysis.

The acidity sites changed remarkably with different metal dosage (Fig. S4a, b† and Table 4). All the Ni–Mg-containing catalysts showed weak + medium and strong acid strength, whereas Ni–Mn containing catalysts exhibited predominantly strongly acid sites. As the amount of Mg increased, weak + medium acidic sites increased remarkably to a maximum of 1815 μmol g^−1^, attributed to basic metal ion (Mg^2+^) that lowered the acid strength of the catalyst.^53^ Accordingly, the acidic strength of Ni–Mg_*n*_/MWCNT reduced upon increased Mg species. Similarly, Ni–Mn-containing catalysts showed a reduction in density of strong acidic sites with the increase in Mn dosage. The basicity of Ni–Mg- and Ni–Mn-containing catalysts are displayed in Fig. S4c, d† and Table 4. All the Ni–Mg-containing catalysts exhibited weak + medium and strong basic sites; meanwhile, the majority of Ni–Mn-containing catalysts exhibited predominantly strongly basic sites. The weakly basic sites in Ni–Mg-containing catalysts indicated the presence of hydroxyl groups on the surface, whereas medium basic sites correlated with the existence of Mg^2+^ and O^2−^. The high basic strength found in all catalysts is due to the isolated O^2−^ species.^54^ Overall, 5 wt% Mg and Mn yielded the highest amount of strongly acidic and basic sites. Thus, these catalysts facilitated cracking instead of the deCOx pathway and increased the formation of light hydrocarbons.^55^

**Fig. 6:**
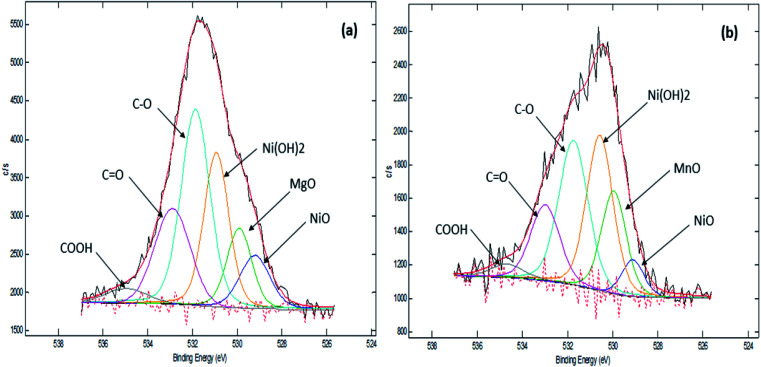
XPS spectra of (a) Ni_10_–Mg_15_/MWCNT and (b) Ni_10_–Mn_5_/MWCNT.

Our previous TPD-CO_2_ analysis (Table 4) showed that Ni_10_–Mg_15_/MWCNT and Ni_10_–Mn_5_/MWCNT produced a high total amount of basic sites with 2162 and 2925 μmol g^−1^, respectively. Other studies have also indicated that basic sites play a major role in promoting decarboxylation by retarding coke formation *via* a decrease in the deactivation rate of acidic catalysts.^56^ Therefore, the XPS characteristics of Ni_10_–Mg_15_/MWCNT and Ni_10_–Mn_5_/MWCNT were examined in our study (Fig. 6). As expected, all the catalysts recorded binding energies (BE) of 284.67, 285.43 and 288.58 eV, which are characteristics of a C species linked to C–C, C–O and C=O. The XPS results proved that the surface of the metal-modified MWCNTs is grafted with oxygen-containing groups.^57^ Indeed, Ni_10_–Mg_15_/MWCNT and Ni_10_–Mn_5_/MWCNT had nickel as Ni^2+^ (529.11 eV, 529.22 eV), magnesium as Mg^2+^ (BE: 529.22 eV) and manganese as Mn^2+^ at BEs of 529.95 eV, respectively. These results were attributed to the existence of NiO, Ni(OH)_2_, MgO and MnO phases, and these findings revealed that an active metal exists in oxide and hydroxide forms.^58^ Overall, the corresponding BE of Ni^2+^ in all binary metal oxide-supported MWCNT catalyst shifts toward a lower energy than that of the corresponding NiO bulk.^59^ A shift in the BE of Ni^2+^ towards lower energies indicates that the metal–support interaction between Ni and MWCNT is strong. A weak metal–support interaction promotes the sintering of Ni and can contribute negatively to the active surface area characteristic under long-term deoxygenation.^60^ This finding suggested that incorporating the Mg and Mn promoters into Ni/MWCNT strongly improves the stability of catalysts during deoxygenation and favours C–O bond cleavage.

**Fig. 7:**
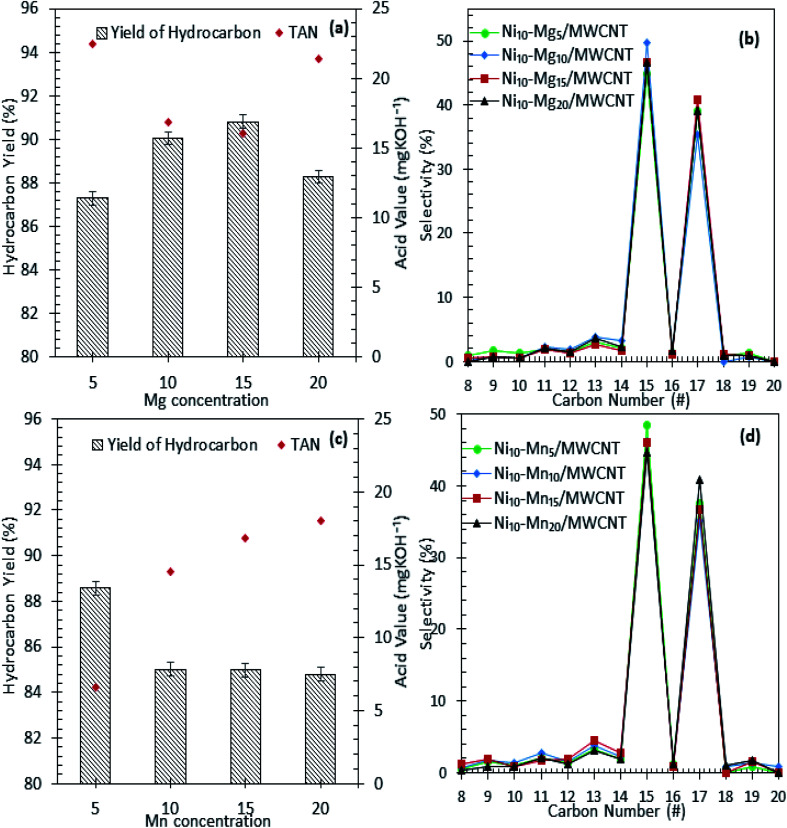
Hydrocarbon yield and product selectivity of deoxygenation reaction (a and b) Ni–Mg_*n*_/MWCNT (c and d) Ni–Mn_*n*_/MWCNT with different Mg and Mn concentration (*n* = 5–20 wt%). Reaction condition: *T* = 350 °C, 2 h reaction time, 3 wt% of catalyst loading.

**Fig. 8:**
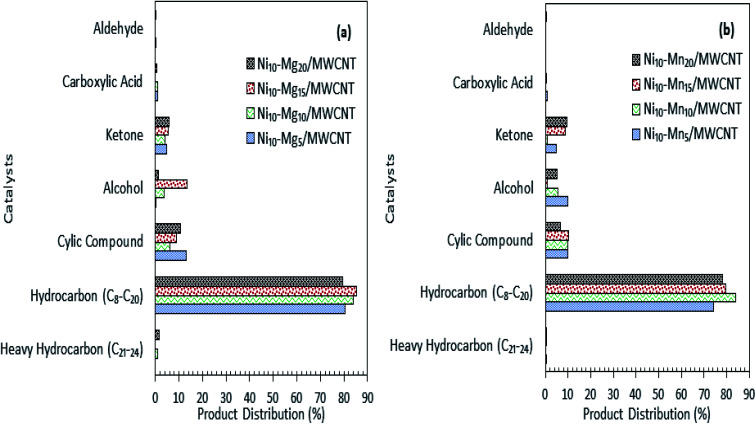
Product distribution of deoxygenated liquid product (a) Ni–Mg_*n*_/MWCNT (b) Ni–Mn_*n*_/MWCNT with different Mg and Mn concentration (*n* = 5–20 wt%).

The results of the deoxygenation activity with different dosages of Mg and Mn on Ni/MWCNT were studied catalytically under the reaction conditions of 350 °C, 2 h and 3 wt% catalyst loading under inert conditions. The results are shown in Fig. 7a–d. It can be observed that the hydrocarbon yield over Ni–Mg-promoted catalysts showed a volcano-shaped curve with respect to the increase in Mg dosage (Fig. 7a). This result indicates that the hydrocarbon fraction is favoured by Mg-species-rich catalysts, yet an excess of Mg species dosage (20 wt%) reduces the hydrocarbon yield, suggesting an increase in C–C bond cleavage of the deoxygenated product *via* cracking.^61^ The maximum hydrocarbon yield (91%) was obtained by Ni_10_–Mg_15_/MWCNT. This result contrasts with the Ni–Mn finding, in which lowest dosage of Mn was shown to result in better deoxygenation performance with a hydrocarbon yield of ∼89% (Fig. 7c). Overall, Ni_10_–Mg_15_/MWCNT rendered more efficient deoxygenation activity than Ni_10_–Mn_5_/MWCNT. A similar trend was observed for *n*-(C_15_ + C_17_) selectivity. The highest *n*-(C_15_ + C_17_) selectivity was reported for Ni_10_–Mg_15_/MWCNT, with a selectivity of 87% (Fig. 7b); meanwhile, Ni_10_–Mn_5_/MWCNT yielded 85% of *n*-(C_15_ + C_17_) (Fig. 7d). It appeared that the catalyst containing 15 wt% Mg species better promoted the deCOx reaction, while it was suppressed by catalysts containing 5 wt% Mn. By comparing the acidity trend of Ni_10_–Mg_15_/MWCNT and Ni_10_–Mn_5_/MWCNT catalysts, it can be inferred that the deCOx reaction is more favoured by catalysts rich in weak + medium acid sites and by the nature of active Mg itself, which induces the decarboxylation pathway.^22^ Parenthetically, the TAN analysis also proved that Ni_10_–Mg_15_/MWCNT rendered the lowest TAN value (16 mgKOH/g). The chemical composition of the liquid product is shown in Fig. 8a and b. It can be seen that the main products were hydrocarbon fractions, cyclic compounds, alcohols and ketones. As expected, Ni_10_–Mg_15_/MWCNT resulted in the highest C_8_–C_20_ hydrocarbon chain (saturated and unsaturated) content (85%). Interestingly, noticeable ketone compounds were detected for Ni_10_–Mg_20_/MWCNT and Ni_10_–Mn_5_/MWCNT, which simultaneously confirmed that ketonisation promotion was facilitated by greater catalyst basicity.

### 3.4 Mass-balance profile for catalytic deoxygenation of CFO

**Fig. 9:**
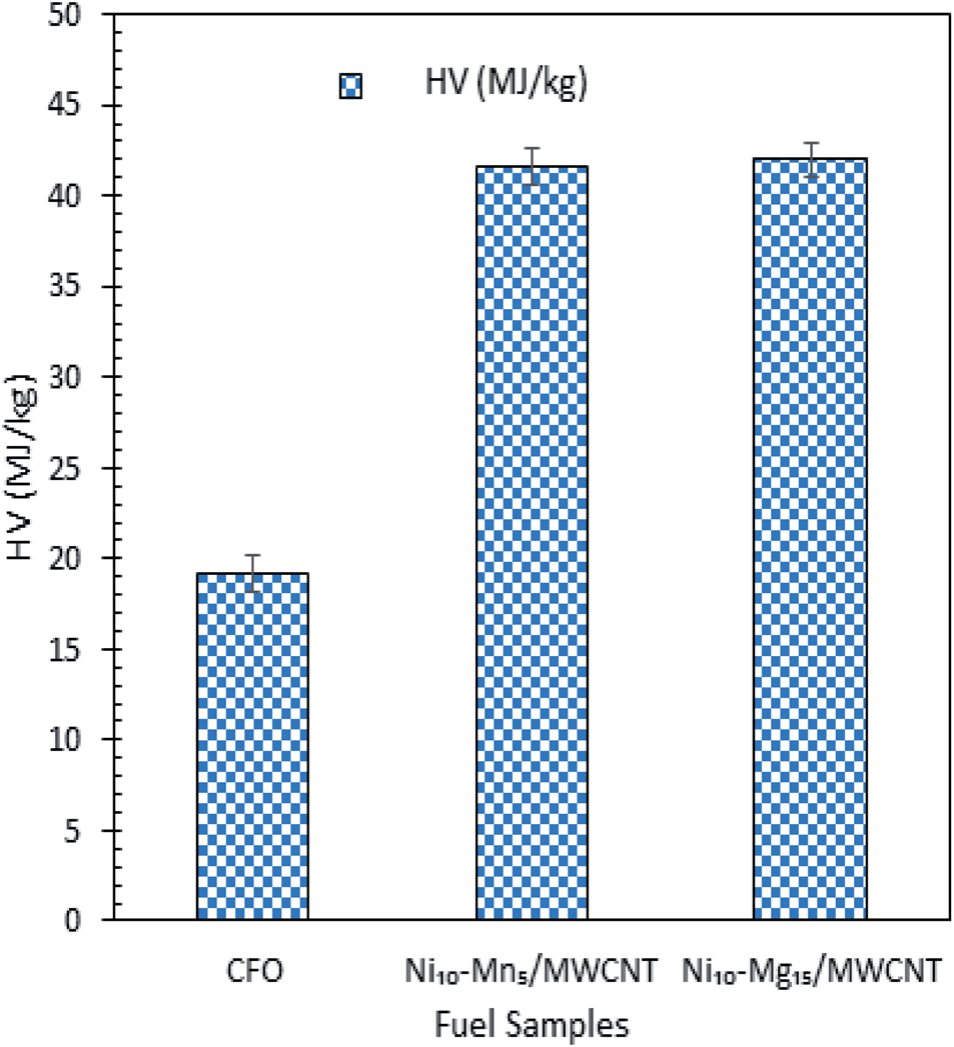
HV test for CFO and green diesel obtained from CFO deoxygenation reaction over Ni_10_–Mg_15_/MWCNT and Ni_10_–Mn_5_/MWCNT catalysts.

The mass-balance profile for catalytic deoxygenation of CFO over a series of Mg and Mn dosages on Ni/MWCNT were determined and results are tabulated in Table 5. Theoretically, CFO will catalytically deoxygenise *via* the deCOx route to produce liquid product along with emission of CO_2_ and CO gases with formation of water as by-product. Based on the results, the experimental mass fraction of liquid product produce from all MWNCT-supported catalysts were within the range of 12–37 wt%. Ni_10_–Mg_15_/MWCNT and Ni_10_–Mn_5_/MWCNT yielded the highest liquid mass fractions, of 37 and 22 wt%, respectively. Regarding the mass fraction of liquid product, the experimental result deviated from the theoretical value, suggesting the formation of undesired char and residue (by-product) produced *via* extensive cracking activity.^62^ Indeed, all deoxygenations showed large amount of gaseous mass fractions (33–55 wt%), which is comparable to the lower liquid product mass fraction. It is worthy of mention that all the catalysed deoxygenation reactions formed negligible amounts of water (<4 wt%). Overall, the mass-balance profile proposed that the most effective and selective catalysts for the deoxygenation of CFO were Ni_10_–Mg_15_ and Ni_10_–Mn_5_, which is in agreement with the above discussion. HV indicates the energy present in a fuel and defines the efficiency of the fuel. Fig. 9 shows the HV of all the green diesels produced by catalysed deoxygenation of CFO over Ni_10_–Mn_5_/MWCNT and Ni_10_–Mg_15_/MWCNT, where the value (41.65–41.98 MJ kg^−1^) is close to ultra-low sulphur diesel (ULSD) specifications (42.5 MJ kg^−1^). Even though the green diesel exhibited lower HV than the standard ULSD, green diesel is a greener biofuel as it produced from products of triglyceride-based biomass and a former study discovered that the green diesel obtained shows greater energy efficiency with lower CO_2_ emissions.^8^ Besides, the product is known to be environmentally friendly and has lower impacts on environment during use than diesel. Thus, the green diesel obtained can be used in diesel engines and is comparable with the ULSD standard.

**Table 5:** Material balance profile of catalytic deoxygenation of CFO

Theoretical deCOx: CFO → liquid (oil) + 3 mol CO_2_/CO (g) + 3 mol H_2_O (aq) + by product									(6)
Reaction^a^	Feedstock (g)	Liq-product^b^		Gas^c^		Water^d^		Char + residue^e^	
		(g)	(wt%)	(g)	(wt%)	(g)	(wt%)	(g)	(wt%)
Theoretical data (deCOx)	10.00	6.89	68.90	2.49	24.90	0.62	6.20	—	—
Ni_10_–Mg_5_	10.00	1.51	15.10	5.06	50.6	0.04	0.40	3.39	33.90
Ni_10_–Mg_10_	10.01	1.51	15.08	4.55	45.45	0.16	1.60	3.79	37.86
Ni_10_–Mg_15_	10.05	3.72	37.01	4.45	44.28	0.01	0.10	1.87	18.61
Ni_10_–Mg_20_	10.02	2.08	20.76	3.78	37.72	0.32	3.19	3.84	38.32
Ni_10_–Mn_5_	10.03	2.22	22.13	5.54	55.23	0.03	0.30	2.24	22.33
Ni_10_–Mn_10_	10.01	1.65	16.48	3.84	38.36	0.07	0.70	4.45	44.46
Ni_10_–Mn_15_	10.01	1.10	10.99	5.09	50.85	0.05	0.50	3.77	37.66
Ni_10_–Mn_20_	10.05	1.24	12.34	3.35	33.33	0.04	0.40	5.42	53.93

a Deoxygenation condition: reaction temperature of 350 °C, 2 h reaction time, 3 wt% of catalyst, under N_2_ environment with stirring at 400 ppm.

b Mass fraction for liq-product = [(mass of liq-product/mass of feedstock) × 100].

c Material fraction for gas = [(mass of feedstock − mass of liq-product − mass of (char + residue) − mass of water)/mass of feedstock × 100].

d Material fraction for water = [(mass of water/mass of feedstock) × 100].

e Material fraction for (char + residue) = [(mass of (char + residue)/mass of feedstock) × 100].

### 3.5 Proposed reaction scheme for catalytic deoxygenation of CFO to diesel-range hydrocarbons over Ni_10_–Mn_5_/MWCNT and Ni_10_–Mg_15_/MWCNT catalysts

**Fig. 10:**
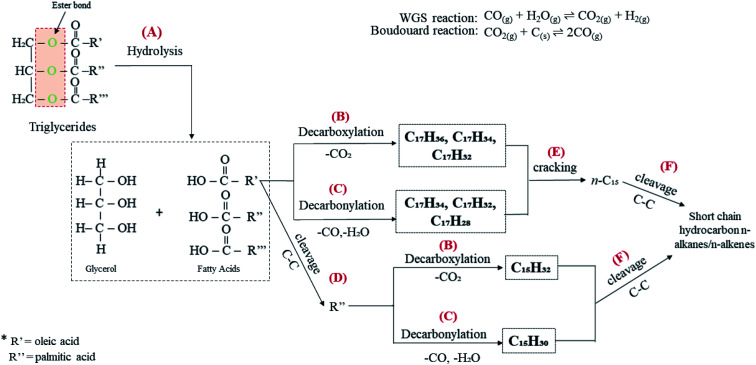
Proposed reaction scheme for catalytic deoxygenation of CFO to hydrocarbon over Ni_10_–Mg_15_/MWCNT and Ni_10_–Mn_5_/MWCNT catalysts.

Table 1 shows that CFO is predominantly composed of long chains of fatty acids, *i.e.* C16 (∼29%) and C18 (∼69%). It is a triglyceride-based oil composed of a triglycerol molecule attached to three fatty acids *via* an ester bond.^63^ Thus, CFO deoxygenation starts with ester hydrolysis, resulting in the breaking down of triglycerides into C16 and C18 fatty acids (reaction A, Fig. 10). Fatty acids are further deoxygenised *via* decarboxylation, forming *n*-heptadecanes (saturated; C_17_H_36_), *n*-heptadecenes (mono-unsaturated, C_17_H_34_; di-unsaturated, C_17_H_32_) and *n*-pentadecanes (saturated, C_15_H_32_; reaction B). This reaction also yields gaseous CO_2_ as a byproduct. Simultaneously, fatty acids undergo decarbonylation *via* the removal of CO and H_2_O as byproducts, thereby producing hydrocarbons with extra double bonds in the form of mono-unsaturated (C_15_H_30_, C_17_H_34_), di-unsaturated (C_17_H_32_) and poly-unsaturated (C_17_H_28_) compounds (reaction C). In our discussion, WGS and Boudouard reactions (eqn (4) and (5)) may occur and produce CO_2_, CO and H_2_. Indeed, the obtained H_2_ should be used for the hydrogenation of unsaturated hydrocarbon to form saturated hydrocarbons.^64^ Thus, the amount of *n*-heptadecanes as products increased (Table S2†). Although the content of C_16_ fatty acids in CFO was 70% lower than that of C_18_ fatty acids, the *n*-C_15_ hydrocarbon fraction dominated in all catalysed reactions. This is probably due to the further cracking of C_18_ fatty acids leading to the formation of C_16_ fatty acid products (reaction D). Furthermore, that cracking of deoxygenated liquid products may also tends to form *n*-C_15_ compounds (reaction E). This phenomenon is in agreement with Kareem's finding in the deoxygenation of waste cooking oil over walnut-shell-derived nanorod-activated carbon supporting Cao-La_2_O_3_, which produced primarily the *n*-C_15_ fraction *via* the deCOx pathway.^33^ Additionally, light hydrocarbons (*n*-(C_8_–C_9_), Fig. 7b and d) were also formed by C–C bond cleavages of the deoxygenated product (reaction F).

### 3.6 Deactivation of the catalyst by coke formation

Typically, deoxygenation catalysts are prone to deactivation due to coke deposition. Therefore, the coke deposited on the spent Ni_10_–Mn_5_/MWCNT and Ni_10_–Mg_15_/MWCNT catalysts were studied using TGA and the results shown in Fig. S5.† The coke formation was determined by the difference in weight loss between the spent and the fresh catalyst.^65^ The spent catalysts were simply reactivated by hexane washing. The result showed that both of the fresh and spent catalysts experienced major weight loss between 350 and 840 °C. The weight loss at 500–650 °C was assigned to the combustion of MWCNT nanorods, while the extra weight loss at 350–499 °C was due to oxidation of the hard coke in air.^66^ In can be seen that both spent Ni_10_–Mg_15_/MWCNT and Ni_10_–Mn_5_/MWCNT catalysts favoured hard coke formation. Hard coke is typically formed at high temperature and has a lower H/C ratio. Interestingly, spent Ni_10_–Mg_15_/MWCNT exhibits two other minor weight losses at <180 °C and 180–330 °C, which can be attributed to the removal of H_2_O + volatile matter (alcohols) (see Fig. 8a) and soft coke. Soft coke is preferentially formed at low reaction temperatures and has high hydrogen-to-carbon ratio and is rich in polyaromatic species,^66^ suggesting that MgO favours polymerisation reactions. In total, spent Ni_10_–Mn_5_/MWCNT catalyst showed higher coke formation (11 wt%) than did Mg-containing catalysts (5 wt%), thereby indicating that Ni_10_–Mg_15_/MWCNT provided greater stability.

### 3.7 Reusability and stability profile for Ni_10_–Mg_15_/MWCNT

**Fig. 11:**
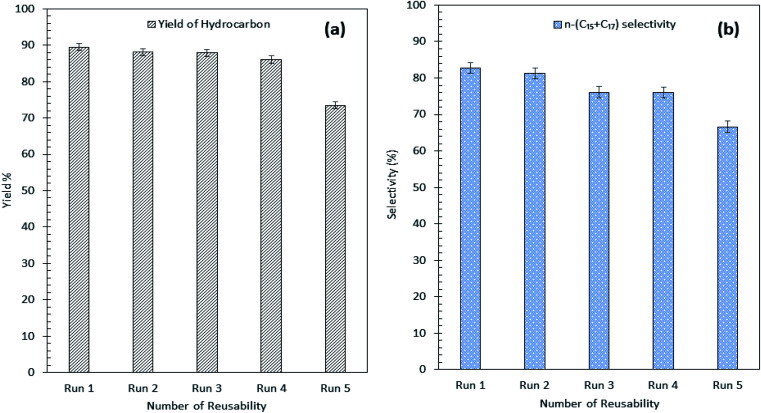
(a) Yield of hydrocarbon and (b) product selectivity of Ni_10_–Mg_15_/MWCNT deoxygenized product from reusability reaction.

**Fig. 12:**
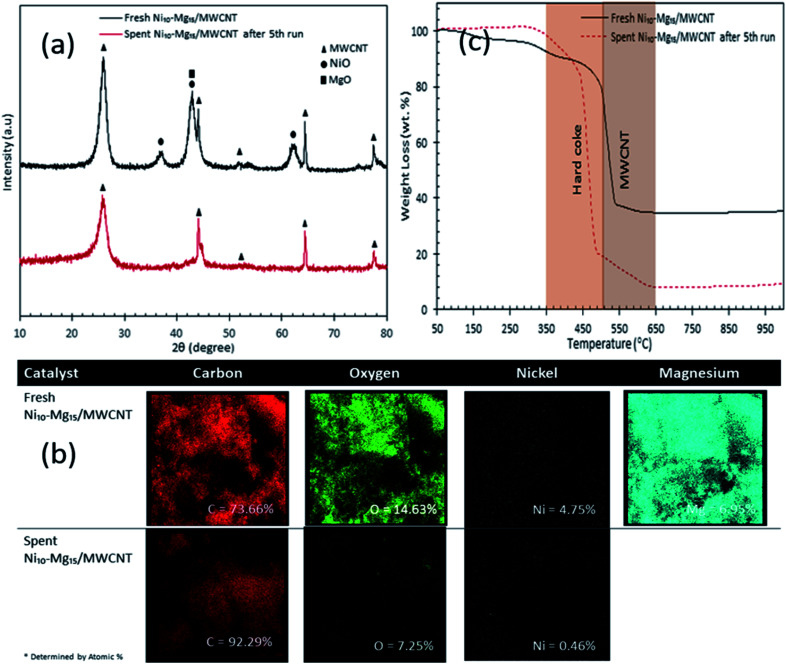
(a) X-ray diffraction patterns, (b) elemental mapping and (c) TGA profile for fresh and spent Ni_10_–Mg_15_/MWCNT catalyst after 5^th^ cycles.

Our findings revealed that Ni_10_–Mg_15_/MWCNT showed high resistancy toward coke formation (coke < 5 wt%). Thus, the reusability and stability of Ni_10_–Mg_15_/MWCNT catalyst were examined using 3 wt% catalyst loading at a reaction temperature of 350 °C within a reaction time of 2 h under inert conditions. After each cycle of deoxygenation was completed, the spent catalyst was reactivated by simply washing with hexane multiple times and reused for the next cycle under the same condition. Hexane washing is the most appropriate technique for reactivating the spent carbon-based catalyst because the nature of the carbon support would preclude the removal of coke deposited on the spent Ni_10_–Mg_15_/MWCNT catalysts *via* a thermal treatment process. Hexane washing has also been commonly practiced to regenerate catalysts because it effectively removes organic species adsorbed on the surface of catalysts and increases the active phase.^67^ Our reusability analysis showed that the Ni_10_–Mg_15_/MWCNT catalyst could be consistently reused by up to five cycles with hydrocarbon yield and *n*-(C_15_ + C_17_) selectivity of 89–73% and 82–66%, respectively (Fig. 11a and b). The deoxygenation activity noticeably decreased at the fifth cycle because of active metal species leaching and coke formation.^68^ This finding agreed with our XRD results. In particular, the cubic structure of NiO at 2*θ*: 37.05°, 43.14° and 62.89° and the hexagonal structure of MgO at 2*θ*: 42.92° disappeared in the spent Ni_10_–Mg_15_/MWCNT catalyst after the fifth cycle (Fig. 12a). This phenomenon might be attributed to the leaching of active metal species in the catalyst. Furthermore, this finding was strongly affirmed by a decrease in the Ni% and the absence of Mg species in FESEM-EDX mapping (Fig. 12b). Nevertheless, the C% on the spent Ni_10_–Mg_15_/MWCNT surfaces also increased to 19% after the fifth cycle, suggesting that coke was deposited on the exterior of the spent catalyst upon deoxygenation. The TGA analysis of the fresh and spent Ni_10_–Mg_15_/MWCNT catalysts (Fig. 12c) showed that both catalysts experienced a major weight loss between 350 °C and 550 °C. The main decomposition peak at 350–499 °C was due to the oxidation of the hard coke in air, whereas the peak at 500–650 °C was attributed to the combustion of MWCNT nanorods. Approximately 26 wt% of hard coke was formed after the fifth cycle. In low-quality feedstock deoxygenation, catalysts remain highly stable in four to six cycles without a noticeable loss of reaction activity,^33^ and the amount of coke formed is minimal (<4 wt%).^69^ The comparison of the performance of their catalysts with that of the Ni_10_–Mg_15_/MWCNT catalyst used in our study indicated that deoxygenation was sensitive to coke formation.

The comparison of the catalytic deoxygenation of various feeds is summarised in Table 6. The results indicated that Ni_10_–Mg_15_/MWCNT showed an excellent deoxygenation activity through which the total hydrocarbon fraction was 90%, and the diesel range selectivity *n*-(C_15_ + C_17_) was 87% compared with that of other catalysts.^16,47,70,71^ Ni- and Mg-supported AC resulted in a low deoxygenation activity through which Ni/AC produced 64–72% hydrocarbon with 51–82% (*n*-C_15_ + C_17_); by comparison, Mg/AC yielded 90% hydrocarbon and ∼50% of *n*-C_15_ + C_17_ selectivity.^16,31,47^ Interestingly, the hydrocarbon yield of Mg/AC was closer to the value obtained *via* the Ni_10_–Mg_15_/MWCNT-catalysed reaction, but the *n*-(C_15_ + C_17_) selectivity was low (∼50%).^31^ This variation was due to the presence of weak + medium acidic sites in Ni_10_–Mg_15_/MWCNT and the high surface area/volume ratio of the Ni_10_–Mg_15_/MWCNT catalyst.^33,69^ This finding agreed with that of Asikin-Mijan *et al.*^30^ who demonstrated that the use of a strongly acidic Ni_20_–Co_10_/MWCNT catalyst produces a high hydrocarbon yield (80%), but the *n*-(C_15_ + C_17_) selectivity of this catalyst is lower than that of Ni_10_–Mg_15_/MWCNT. This finding strongly affirmed that weakly or moderately acidic sites in Ni_10_–Mg_15_/MWCNT catalyst promoted C–O bond cleavage to a greater extent than strong acidic sites in Ni_20_–Co_10_/MWCNT catalyst did. Another study has also shown that H_2_ is beneficial to the hydrogenation of an unsaturated species to a saturated compound and directly prevents the unsaturated species from deactivating the active sites of catalysts.^72^ However, the use of H_2_ could induce a competitive reaction between deCOx and hydrogenation, thus decreasing the catalytic activity. Evidently, Kaewmeesri and co-worker studied the deoxygenation of CFO under a H_2_ atmosphere but only obtained 72% of hydrocarbon and 82% *n*-(C_15_ + C_17_) selectivity compared with that of Ni_10_–Mg_15_/MWCNT-catalysed CFO.^16^ The presence of H_2_ results in limited surface active sites for hydrogen and feedstock molecules, concurrently decreasing the deCOx reaction.^73^ In conclusion, a reaction in a H_2_-free environment provided an effective condition for an efficient deoxygenation activity to produce high-quality green diesel.

**Table 6:** Comparison study on catalytic deoxygenation of various of feeds

No.	Catalyst	Support	Reaction	Feed	Hydrocarbon yield (%)	Selectivity (%)	References
1	10%Mg/AC^a^	AC^a^ (commercial)	DO	WCO^b^	90	<50 (*n*-C_15_ + C_17_)	70
2	20%Ni/C	AC^a^ (commercial)	DO H_2_ (39 bar)	Stearic acid	64	51 (*n*-C_15_ + C_17_)	47
3	Ni/γ-Al_2_O_3_	γ-Al_2_O_3_ (commercial)	DO H_2_ (50 bar)	CFO	72	82 (*n*-C_15_ + C_17_)	16
4	Ni_20_–Co_10_/MWCNT	MWCNT (commercial)	DO	JCO^c^	80	64 (*n*-C_15_ + C_17_)	46
5	Ni_10_–Mg_15_/MWCNT	MWCNT (commercial)	DO	CFO	90	87 (*n*-C_15_ + C_17_)	Present study

a AC = activated carbon.

b WCO = waste cooking oil.

c JCO = *Jatropha curcas* oil.

## 4. Conclusions

In the present work, oxides of binary metal pairs (Ni–Mg, Ni–Mn, Ni–Cu, Ni–Ce) supported on MWCNTs showed promising catalytic activity in improving the deoxygenation of CFO for the formation of diesel-like hydrocarbons, yielding 72–84% of *n*-(C_8_–C_20_) hydrocarbon fractions with a product selectivity towards *n*-(C_15_ + C_17_) of 59–84%. In total, the binary Ni_10_–Mg_10_/MWCNT and Ni_10_–Mn_10_/MWCNT catalysts rendered highest deoxygenation activity favourable towards the deCOx reaction. The carbon number range of hydrocarbons produced is pragmatically tuneable by varying the active metal concentrations. Mg-rich species (15 wt%) and low-Mn species (5 wt%) afforded greater deoxygenation performance *via* deCOx pathways, with product selectivities towards *n*-(C_15_ + C_17_) of 87% and 85%, respectively. The high reactivity of Ni_10_–Mg_15_/MWCNT was due mainly to the presence of weak + medium acid sites and to the nature of active Mg itself, which induced the decarboxylation pathway. Ni_10_–Mg_15_/MWCNT showed greater stability than Ni_10_–Mn_5_/MWCNT, suggested due to the presence of coke inhibitor promoter (MgO).

## Conflicts of interest

There are no conflicts to declare.

## Supplementary Material

RA-010-C9RA08409F-s001

## References

[cit1] Gurunathan B., Ravi A. (2015). Bioresour. Technol..

[cit2] Bezergianni S., Dimitriadis A., Sfetsas T., Kalogianni A. (2010). Bioresour. Technol..

[cit3] Gosselink R. W., Hollak S. A. W., Chang S. W., Van Haveren J., De Jong K. P., Bitter J. H., Van Es D. S. (2013). ChemSusChem.

[cit4] Wang W., Qiao Z., Zhang K., Liu P., Yang Y., Wu K. (2014). RSC Adv..

[cit5] Asikin-Mijan N., Lee H. V., Marliza T. S., Taufiq-Yap Y. H. (2018). J. Anal. Appl. Pyrolysis.

[cit6] Echaroj S., Sahasakmontri T., Suntikunaporn M. (2015). Int. Proc. Chem., Biol. Environ. Eng..

[cit7] Asikin-Mijan N., Lee H. V., Taufiq-Yap Y. H., Abdulkrem-Alsultan G., Mastuli M. S., Ong H. C. (2017). Energy Convers. Manage..

[cit8] Douvartzides S. L., Charisiou N. D., Papageridis K. N., Goula M. A. (2019). Energies.

[cit9] Khan T. M. Y., Atabani A. E., Badruddin I. A., Badarudin A., Khayoon M. S., Triwahyono S. (2014). Renewable Sustainable Energy Rev..

[cit10] Mutreja V., Singh S., Ali A. (2011). Renewable Energy.

[cit11] Boey P. L., Maniam G. P., Hamid S. A., Ali D. M. H. (2011). J. Am. Oil Chem. Soc..

[cit12] Jeong G. T., Yang H. S., Park D. H. (2009). Bioresour. Technol..

[cit13] Hoque M. E., Singh A., Chuan Y. L. (2011). Biomass Bioenergy.

[cit14] Seidavi A. R., Zaker-Esteghamati H., Scanes C. G. (2019). World's Poult. Sci. J..

[cit15] Alptekin E., Canakci M., Sanli H. (2014). Waste Manag..

[cit16] Kaewmeesri R., Srifa A., Itthibenchapong V., Faungnawakij K. (2015). Energy Fuels.

[cit17] Yang Y., Wang Q., Zhang X., Wang L., Li G. (2013). Fuel Process. Technol..

[cit18] Onyestyák G., Harnos S., Szegedi Á., Kalló D. (2012). Fuel.

[cit19] Şenol O. I., Ryymin E. M., Viljava T. R., Krause A. O. I. (2007). J. Mol. Catal. A: Chem..

[cit20] Morgan T., Grubb D., Santillan-Jimenez E., Crocker M. (2010). Top. Catal..

[cit21] Rezgui Y., Guemini M. (2005). Appl. Catal., A.

[cit22] Tani H., Hasegawa T., Shimouchi M., Asami K., Fujimoto K. (2011). Catal. Today.

[cit23] Aysu T., Maroto-Valer M. M., Sanna A. (2016). Bioresour. Technol..

[cit24] Loe R., Santillan-Jimenez E., Morgan T., Sewell L., Ji Y., Jones S., Isaacs M. A., Lee A. F., Crocker M. (2016). Appl. Catal., B.

[cit25] Asikin-Mijan N., Taufiq-Yap Y. H., Lee H. V. (2015). Chem. Eng. J..

[cit26] Uddin M. N., Daud W. M. A. W., Abbas H. F. (2014). RSC Adv..

[cit27] Murali Dhar G., Srinivas B. N., Rana M. S., Kumar M., Maity S. K. (2003). Catal. Today.

[cit28] Zhao C., Brück T., Lercher J. A. (2013). Green Chem..

[cit29] Zhang H., Lancelot C., Chu W., Hong J., Khodakov A. Y., Chernavskii P. A., Zheng J., Tong D. (2009). J. Mater. Chem..

[cit30] Asikin-Mijan N., Lee H. V., Abdulkareem-Alsultan G., Afandi A., Taufiq-Yap Y. H. (2018). J. Cleaner Prod..

[cit31] Abdulkareem-Alsultan G., Asikin-Mijan N., Mansir N., Lee H. V., Zainal Z., Islam A., Taufiq-Yap Y. H. (2019). J. Anal. Appl. Pyrolysis.

[cit32] Kalantar E., Kabir K., Gharibi F., Hatami S., Maleki A. (2013). J. Med. Bacteriol..

[cit33] Alsultan G. A., Asikin-Mijan N., Lee H. V., Albazzaz A. S., Taufiq-Yap Y. H. (2017). Energy Convers. Manage..

[cit34] Ang B. C., Yaacob I. I., Nurdin I. (2013). J. Nanomater..

[cit35] Wu J., Xia Q., Wang H., Li Z. (2014). Appl. Catal., B.

[cit36] Rodríguez L. A. A., Pianassola M., Travessa D. N. (2017). Mater. Res..

[cit37] Bom D., Andrews R., Jacques D., Anthony J., Chen B., Meier M. S., Selegue J. P. (2002). Nano Lett..

[cit38] Mirghiasi Z., Bakhtiari F., Darezereshki E., Esmaeilzadeh E. (2014). J. Ind. Eng. Chem..

[cit39] Ponminiessary R. P. , Stud. Prep. Support. Nickel Catal. Using Bis(Ethylenediamine) Nickel(II) Complexes as Precursors, 2013, vol. 1–32

[cit40] Olusola J. O., Adediran M. M., Oluseyi A. K., Ajao U. L. (2010). Energy Environ..

[cit41] Wan Y., Zhao W., Tang Y., Li L., Wang H., Cui Y., Gu J., Li Y., Shi J. (2014). Appl. Catal., B.

[cit42] Wang W., Zhang C., Chen G., Zhang R. (2019). Appl. Sci..

[cit43] Jothi Thirumal B., James Gunasekaran E., Loganathan, Saravanan C. G. (2015). J. Eng. Sci. Technol..

[cit44] Mierczynski P., Mosinska M., Zakrzewski M., Dawid B., Ciesielski R., Maniukiewicz W., Maniecki T. (2017). React. Kinet., Mech. Catal..

[cit45] Baharudin K. B., Taufiq-Yap Y. H., Hunns J., Isaacs M., Wilson K., Derawi D. (2019). Microporous Mesoporous Mater..

[cit46] Asikin-Mijan N., Lee H. V., Juan J. C., Noorsaadah A. R., Taufiq-Yap Y. H. (2017). RSC Adv..

[cit47] Santillan-Jimenez E., Morgan T., Shoup J., Harman-Ware A. E., Crocker M. (2014). Catal. Today.

[cit48] Renz M. (2005). Eur. J. Org. Chem..

[cit49] Putra R., Lestari W. W., Wibowo F. R., Susanto B. H. (2018). Bull. Chem. React. Eng. Catal..

[cit50] Sharifvaghefi S., Zheng Y. (2018). Can. J. Chem. Eng..

[cit51] Gardner D. C., Bartholomew C. H. (1981). Ind. Eng. Chem. Prod. Res. Dev..

[cit52] Biswas P., Kunzru D. (2007). Int. J. Hydrogen Energy.

[cit53] Gabrovska M. , Bankova M., Idakiev V., Edreva-Kardjieva R. and Uzunov I., in Proceeding of the 9th International Symposium of Heterogeneous Catalysis, 2000, pp. 513–518

[cit54] Hu J., Zhu K., Chen L., Kübel C., Richards R. (2007). J. Phys. Chem. C.

[cit55] Krobkrong N., Itthibenchapong V., Khongpracha P., Faungnawakij K. (2018). Energy Convers. Manage..

[cit56] Si Z., Zhang X., Wang C., Ma L., Dong R. (2017). Catalysts.

[cit57] Akalework N. G., Pan C.-J., Su W.-N., Rick J., Tsai M.-C., Lee J.-F., Lin J.-M., Tsai L.-D., Hwang B.-J. (2012). J. Mater. Chem..

[cit58] Castaño P., Pawelec B., Fierro J. L. G., Arandes J. M., Bilbao J. (2007). Fuel.

[cit59] Liu J., He J., Wang L., Li R., Chen P., Rao X., Deng L., Rong L., Lei J. (2016). Sci. Rep..

[cit60] Lou Y., He P., Zhao L., Song H. (2016). Fuel.

[cit61] Kim I., Dwiatmoko A. A., Choi J. W., Suh D. J., Jae J., Ha J. M., Kim J. K. (2017). J. Ind. Eng. Chem..

[cit62] Vitolo S., Seggiani M., Frediani P., Ambrosini G., Politi L. (1999). Fuel.

[cit63] Sotelo-Boyas R. , Trejo-Zarraga F. and de Jesus Hernandez-Loyo F., Hydrogenation, 2012, ch. 8, pp. 187–215

[cit64] Zulkepli S., Juan J. C., Lee H. V., Rahman N. S. A., Show P. L., Ng E. P. (2018). Energy Convers. Manage..

[cit65] Asplund S. (1996). J. Catal..

[cit66] Sahoo S. K., Ray S. S., Singh I. D. (2004). Appl. Catal., A.

[cit67] Wahyudi A., Kurniawan W., Hinode H. (2017). Green Sustainable Chem..

[cit68] Istadi I., Mabruro U., Kalimantini B. A., Buchori L., Anggoro D. D. (2016). Bull. Chem. React. Eng. Catal..

[cit69] Abdulkareem-alsultan G., Asikin-mijan N., Mansir N., V Lee H., Zainal Z. (2018). J. Anal. Appl. Pyrolysis.

[cit70] Natewonga P., Murakamib Y., Tanic H., Asami K. (2016). Am. Sci. Res. J. Eng. Technol. Sci..

[cit71] Asikin-mijan N., Lee H. V., Abdulkareem-alsultan G., Afandi A. (2017). J. Cleaner Prod..

[cit72] Srifa A., Faungnawakij K., Itthibenchapong V., Assabumrungrat S. (2015). Chem. Eng. J..

[cit73] Wang W. C., Thapaliya N., Campos A., Stikeleather L. F., Roberts W. L. (2012). Fuel.

