# Supplementary material for: Production of green diesel from catalytic deoxygenation of chicken fat oil over a series binary metal oxide-supported MWCNTs
Source: RSC Adv. 2020 Jan 2;10(2):626–42. doi: 10.1039/c9ra08409f (PMC9047115; doi:10.1039/c9ra08409f)
Supplement: RA-010-C9RA08409F-s001 [file RA-010-C9RA08409F-s001.pdf]

This journal is © The Royal Society of Chemistry 2019

List of Supplementary Table

Table S1. GC-MS concentration (wt %) of the deoxygenated liquid product

Table S2. GC-MS concentration (wt %) of the deoxygenated product catalysed by Ni_{10}-Mg_{15}/MWCNT and Ni_{10}-Mn_5/MWCNT

Table S1. GC-MS concentration (wt.%) of the deoxygenated liquid product

Catalyst	Compound group	Compound name	Concentration (%)
MWCNT	Carboxylic acid	n-Hexadecanoic acid	1.278
		Trifluoroacetic acid, pentadecyl ester	0.367
	Ketone	2-Nonadecanone	0.909
	Alcohol	n-Tetracosanol-1	1.205
		1-Heptacosanol	2.635
		9-Octadecanol, (E)-	0.168
		9-Hexadecanol, (E)-	0.212
	Cylic Hydrocarbon (C_8-C_{20})	Cyclopropane	3.769
		n-Nonylcyclohexane	1.454
		Cyclohexene	0.586
		Cyclopentane	0.592
		Bicyclohexyl	0.324
		Cyclohexane	0.221
	Hydrocarbon (C_8-C_{20})	**<u>Saturated Hydrocarbon</u>**	
		Nonane	3.845
		Undecane	2.046
		Dodecane	2.376
		Tridecane	4.806
		Tetradecane	5.294
		Pentadecane	31.508
		Hexadecane	1.927
		Heptadecane	0.269
		**<u>Unsaturated Hydrocarbon</u>**	
		1-Nonene	
		1-Decene	0.726
		1-Undecene	0.661
		1-Tridecene	0.996
		1-Tetradecene	1.389
		7-Tetradecene	1.901
		9-Octadecene, (E)-	
		1-Pentadecene	2.061
		5-Eicosene, (E)-	1.362
		3-Heptadecene, (Z)-	0.247
		1-Octadecene	6.895
		7-Heptadecene	0.283
		9-Eicosene, (E)-	0.269
			2.094
	Heavy Hydrocarbon	Hexacosane	0.380
		9-Tricosene, (Z)-	1.707

	(C_{21}-C_{24})		
Ni_{10} / MWCNT	Carboxylic acid	Trifluoroacetic acid, pentadecyl ester	1.284
	Alcohol	1-Undecanol	3.782
	Cylic Hydrocarbon (C_8-C_{20})	Cyclopropane<br>Benzene, pentyl-<br>Cyclopentane<br>Cyclododecene, (Z)-<br>Benzene, (1-methyldecyl)-<br>n-cyclohexane	2.272<br>1.939<br>1.069<br>0.614<br>1.413<br>1.540
	Hydrocarbon (C_8-C_{20})	**Saturated Hydrocarbon**<br>Octane<br>Nonane<br>Undecane<br>Dodecane<br>Tetradecane<br>Hexadecane<br>Heptadecane<br><br>**Unsaturated Hydrocarbon**<br>1-Nonene<br>1-Decene<br>1-Undecene<br>3-Dodecene, (E)-<br>1-Tridecene<br>1-Tetradecene<br>7-Tetradecene<br>1-Hetptadecene<br>9-Octadecene, (E)-<br>3-Heptadecene, (Z)-<br>5-Heptadecene	 1.056<br>3.206<br>2.815<br>3.001<br>8.395<br>7.963<br>14.109<br><br><br>0.696<br>0.951<br>1.797<br>1.997<br>1.366<br>0.955<br>7.419<br>4.512<br>0.861<br>15.028<br>0.675
Ni_{10}–Mn_{10}/ MWCNT	Ketone	2-Nonadecanone	0.748
	Alcohol	1-Undecanol<br>n-Tridecan-ol	3.536<br>1.915
	Cylic Hydrocarbon (C_8-C_{20})	Cyclopentene,<br>Benzene, pentyl-<br>Cyclopropane<br>n-Nonylcyclohexane<br>Cyclopentane<br>Cyclododecanemethanol	2.35<br>1.507<br>1.024<br>0.948<br>0.702<br>0.474
	Hydrocarbon (C_8-C_{20})	**Saturated Hydrocarbon**<br>Nonane<br>Undecane<br>Dodecane<br>Tetradecane<br>Hexadecane<br>Heptadecane	 1.005<br>2.165<br>1.639<br>5.180<br>35.07<br>9.032

		**Unsaturated Hydrocarbon**<br>1-Decene<br>Trans-3-Decene<br>1-Undecene<br>3-Dodecene, (E)-<br>1-Tridecene<br>1-Tetradecene<br>9-Octadecene, (E)-<br>1-Pentadecene<br>3-Heptadecene, (Z)-<br>5-Heptadecene<br>9-Eicosene, (E)-	 0.857<br>0.747<br>1.560<br>0.440<br>0.750<br>0.651<br>2.625<br>1.961<br>10.807<br>0.829<br>0.150
Ni_{10}–Mg_{10}/ MWCNT	Carboxylic acid	Dichloroacetic acid<br>Trifluoroacetic acid	**0.918**<br>**0.370**
	Ketone	9-Octadecanone<br>2-Pentadecanone<br>3-Octadecanone<br>8-Octadecanone<br>9-Heptadecanone<br>10-Nonadecanone	0.319<br>1.716<br>0.387<br>0.452<br>0.508<br>0.318
	Alcohol	1-Undecanol<br>1-Dodecanol<br>Octacosanol<br>1-Heptacosanol	1.792<br>0.800<br>0.557<br>1.405
	Cylic Hydrocarbon (C_8-C_{20})	Cyclopropane<br>n-Nonylcyclohexane<br>Cyclohexene<br>Cyclopentane<br>Cyclohexane<br>Cyclohexadecane	0.952<br>1.455<br>0.474<br>1.136<br>0.894<br>11.490
	Hydrocarbon (C_8-C_{20})	**Saturated Hydrocarbon**<br>Nonane<br>Undecane<br>Dodecane<br>Tetradecane<br>Hexadecane<br>Heptadecane<br><br>**Unsaturated Hydrocarbon**<br>1-Decene<br>1-Undecene<br>1-Tridecene<br>1-Tetradecene<br>7-Tetradecene<br>9-Octadecene, (E)-<br>1-Pentadecene<br>9-Eicosene, (E)-	 1.121<br>1.122<br>1.742<br>4.641<br>35.348<br>11.490<br><br><br>0.370<br>0.802<br>0.810<br>0.810<br>1.371<br>2.807<br>0.587<br>1.037

		5-Eicosene, (E)-<br>3-Heptadecene, (Z)-<br>5-Eicosyne	0.126<br>10.466<br>0.279
	Heavy Hydrocarbon (C_{21}-C_{24})	9-Tricosene, (Z)-	1.005
Ni_{10}–Ce_{10}/ MWCNT	Carboxylic acid	Malonic acid	0.617
	Ketone	2-Nonadecanone	0.612
	Alcohol	1-Undecanol<br>1-Dodecanol<br>n-Pentadecanol<br>n-Tetracosanol	2.205<br>0.814<br>4.810<br>0.516
	Cylic Hydrocarbon (C_8-C_{20})	Cyclopropane<br>Heptane, 1,1-dicyclohexyl<br>n-Nonylcyclohexane<br>Cyclopentane	0.771<br>0.450<br>1.143<br>1.25
	Hydrocarbon (C_8-C_{20})	**Saturated Hydrocarbon**<br>Octane<br>Nonane<br>Undecane<br>Dodecane<br>Tetradecane<br>Hexadecane<br>Heptadecane<br><br>**Unsaturated Hydrocarbon**<br>1-Nonene<br>1-Decene<br>2-Decene<br>1-Undecene<br>1-Tetradecene<br>7-Tetradecene<br>9-Octadecene, (E)-<br>1-Pentadecene<br>5-Octadecene, (E)-<br>3-Heptadecene, (Z)-<br>5-Heptadecene	 2.255<br>6.671<br>2.466<br>1.423<br>4.373<br>35.564<br>10.995<br><br><br>1.196<br>0.761<br>0.526<br>0.950<br>0.631<br>1.278<br>2.269<br>2.568<br>0.436<br>3.780<br>0.463
Ni_{10}–Cu_{10}/ MWCNT	Carboxylic acid	Nonanoic acid	**0.046**
	Ketone	2-Nonadecanone	0.462
	Alcohol	1-Undecanol<br>1-Dodecanol<br>n-Heptadecanol	2.189<br>0.993<br>2.958

	Cylic Hydrocarbon (C_8-C_{20})	Cyclopentene<br>Benzene<br>Cyclopropane<br>Cyclopentane<br>n-Pentadecyclohexane<br>Cyclohexene<br>Cyclopentane<br>Cyclohexane	1.240<br>1.113<br>0.786<br>0.588<br>1.017<br>0.227<br>0.631<br>0.441
	Hydrocarbon (C_8-C_{20})	**Saturated Hydrocarbon**<br>Octane<br>Nonane<br>Undecane<br>Dodecane<br>Tridecane<br>Tetradecane<br>Hexadecane<br><br>**Unsaturated Hydrocarbon**<br>1-Octene<br>1-Nonene<br>2-Nonene, (E)-<br>1-Decene<br>2-Decene, (E)-<br>1-Undecene<br>1-Tridecene<br>7-Tetradecene<br>9-Octadecene, (E)-<br>1-Pentadecene<br>9-Eicosene, (E)-<br>3-Heptadecene, (Z)-	 0.505<br>10.177<br>6.03<br>2.125<br>4.547<br>3.431<br>29.845<br><br><br>0.505<br>1.686<br>0.861<br>0.976<br>0.617<br>1.112<br>1.031<br>0.294<br>1.367<br>1.901<br>0.972<br>0.972
	Heavy Hydrocarbon (C_{21}-C_{24})	Tetracosane<br>9-Tricosene,(Z)-<br>Heneicosane	0.706<br>0.656<br>0.630

Table S2. GC-MS concentration (wt.%) of the deoxygenated product catalysed by Ni_{10}-Mg_{15}/MWCNT and Ni_{10}-Mn_5/MWCNT

Catalyst	Compound group	Compound name	Concentration (%)
Ni_{10}-Mg_{15}/ MWCNT	Ketone	2-Pentadecanone<br>3-Octadecanone<br>2-Nonadecanone<br>9-Heptadecanone<br>10-Nonadecanone	3.215<br>0.692<br>0.714<br>0.498<br>0.241
	Alcohol	n-Pentadecanol<br>Oleyl alcohol<br>Z-(13,14-Epoxy)tetradec-11-en-1-ol acetate<br>1-Heneicosanol<br>Octacosanol	10.040<br>1.575<br>0.745<br>0.069<br>0.780
	Cylic Hydrocarbon (C_8-C_{20})	Cyclopropane<br>n-Nonylcyclohexane<br>Cyclohexene<br>Cyclopentane<br>Cyclohexane	4.267<br>1.498<br>0.511<br>1.146<br>0.567
	Hydrocarbon (C_8-C_{20})	**Saturated Hydrocarbon**<br>Nonane<br>Undecane<br>Dodecane<br>Tridecane<br>Tetradecane<br>Hexadecane<br>Heptadecane<br>Octadecane<br><br>**Unsaturated Hydrocarbon**<br>1-Nonene<br>1-Decene<br>1-Undecene<br>1-Tridecene<br>1-Tetradecene<br>7-Tetradecene<br>9-Octadecene, (E)-<br>1-Pentadecene<br>5-Eicosene, (E)-<br>3-Heptadecene, (Z)-<br>5-Octadecene, (E)-<br>5-Heptadecene<br>9-Eicosene, (E)-	 1.840<br>3.944<br>1.927<br>5.862<br>3.865<br>9.222<br>16.427<br>0.751<br><br><br>0.461<br>0.550<br>1.008<br>1.603<br>1.060<br>2.347<br>5.638<br>2.539<br>0.326<br>10.368<br>0.346<br>0.412<br>0.287
Ni_{10}-Mn_5/	Carboxylic acid	Malanoic acid, 2-hepthyl tetradecyl ester	0.761

MWCNT	Ketone	2-Pentadecanone<br>3-Octadecanone<br>2-Nonadecanone<br>8-Pentadecanone<br>9-Heptadecanone<br>10-Nonadecanone	2.950<br>0.515<br>0.609<br>0.318<br>0.324<br>0.183
	Alcohol	n-Pentadecanol<br>n-Nonadecanol-1<br>1-Heneicosanol<br>Octacosanol	8.836<br>0.413<br>0.156<br>0.452
	Cylic Hydrocarbon (C_8-C_{20})	Furan, 2-butyltetrahydro-<br>Cyclopropane<br>n-Nonylcyclohexane<br>Cyclohexene<br>Bicyclohexyl<br>Cyclohexane<br>Benzene, (1-methyldecyl)-	1.352<br>4.473<br>1.396<br>0.463<br>0.472<br>0.592<br>1.255
	Hydrocarbon (C_8-C_{20})	<u>**Saturated Hydrocarbon**</u><br>Octane<br>Nonane<br>Undecane<br>Dodecane<br>Tridecane<br>Tetradecane<br>Hexadecane<br>Heptadecane<br>Nonadecane<br><br><u>**Unsaturated Hydrocarbon**</u><br>1-Nonene<br>1-Decene<br>2-Decene, (E)-<br>1-Undecene<br>1-Tridecene<br>1-Tetradecene<br>9-Octadecene, (E)-<br>1-Pentadecene<br>5-Octadecene, (E)-<br>5-Eicosene, (E)-<br>3-Heptadecene, (Z)-<br>5-Octadecene, (E)-<br>5-Heptadecene<br>9-Octadecene, (E)-	 1.031<br>2.797<br>4.516<br>1.917<br>5.500<br>3.080<br>11.149<br>15.967<br>0.645<br><br><br>0.690<br>0.725<br>0.494<br>1.082<br>0.826<br>0.956<br>6.071<br>4.342<br>0.634<br>1.102<br>8.544<br>0.402<br>0.712<br>0.243
	Heavy	1-Docosene	0.203

	Hydrocarbon (C_{21}-C_{24})		

List of Supplementary Data

Fig. S1. TGA profile for the MWCNT and MWCNT supported catalysts.

Fig. S2. (a) TPD-NH_3 analysis and (b) TPD-CO_2 profile for the MWCNT and MWCNT supported catalysts.

Fig. S3. X-ray diffraction for (a) Ni-Mg_n/MWCNT (b) Ni-Mn_n/MWCNT with different Mg and Mn concentration (n=5-20 wt %).

Fig. S4. TPD-NH_3 analysis of (a) Ni-Mg_n/MWCNT (b) Ni-Mn_n/MWCNT and TPD-CO_2 (c) Ni-Mg_n/MWCNT (d) Ni-Mn_n/MWCNT with different Mg and Mn concentration (n=5-20 wt %).

Fig. S5. TGA for fresh and spent (a) Ni_{10}-Mg_{15}/MWCNT and (b) Ni_{10}-Mn_5/MWCNT catalysts.

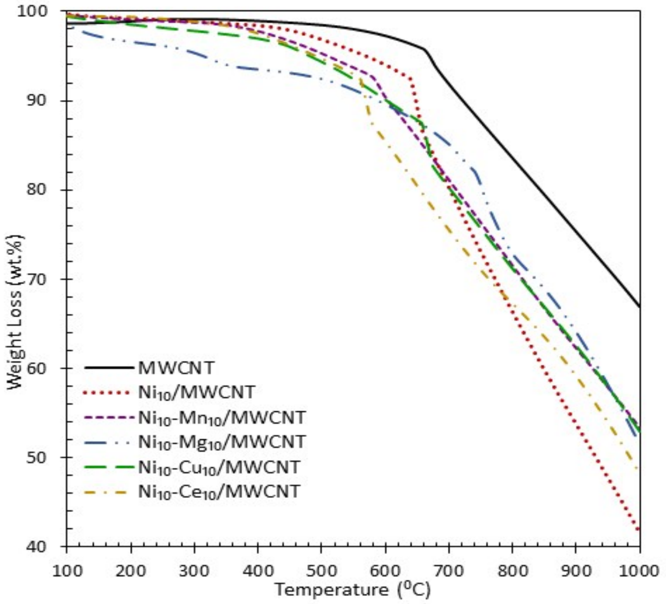

Fig. S1. TGA profile for the MWCNT and MWCNT supported catalysts.

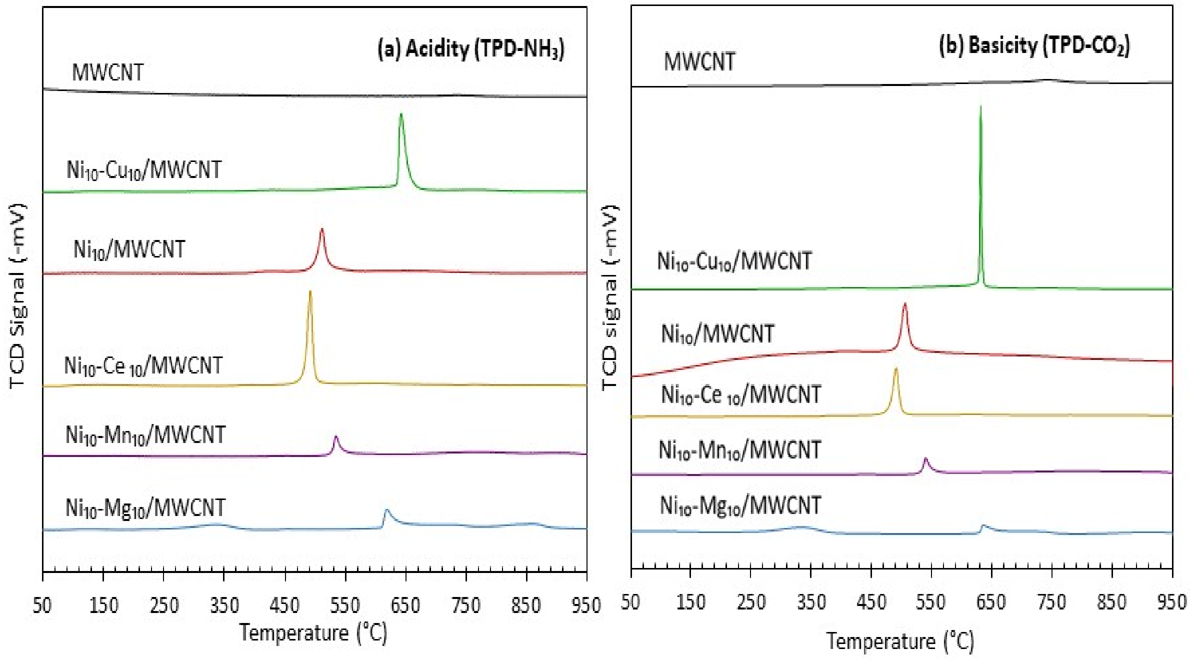

Fig. S2. (a) TPD-NH_3 analysis and (b) TPD-CO_2 profile for the MWCNT and MWCNT supported catalysts.

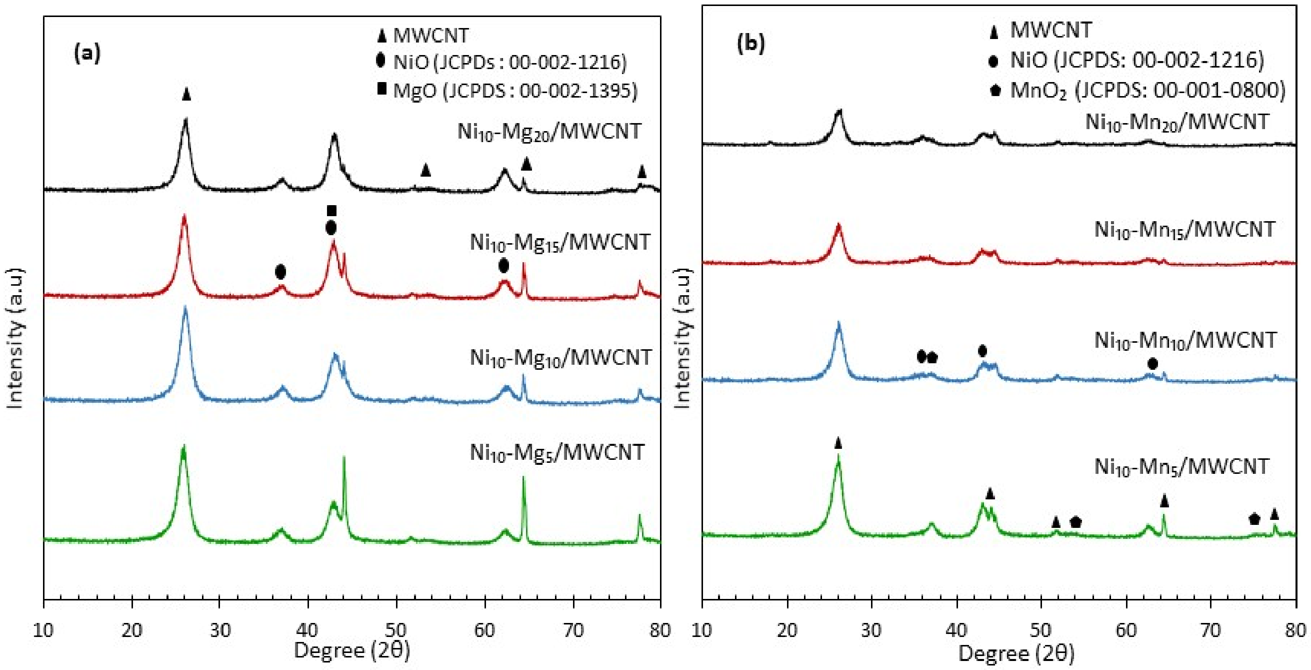

Fig. S3. X-ray diffraction for (a) Ni-Mg_n/MWCNT (b) Ni-Mn_n/MWCNT with different Mg and Mn concentration (n=5-20 wt %).

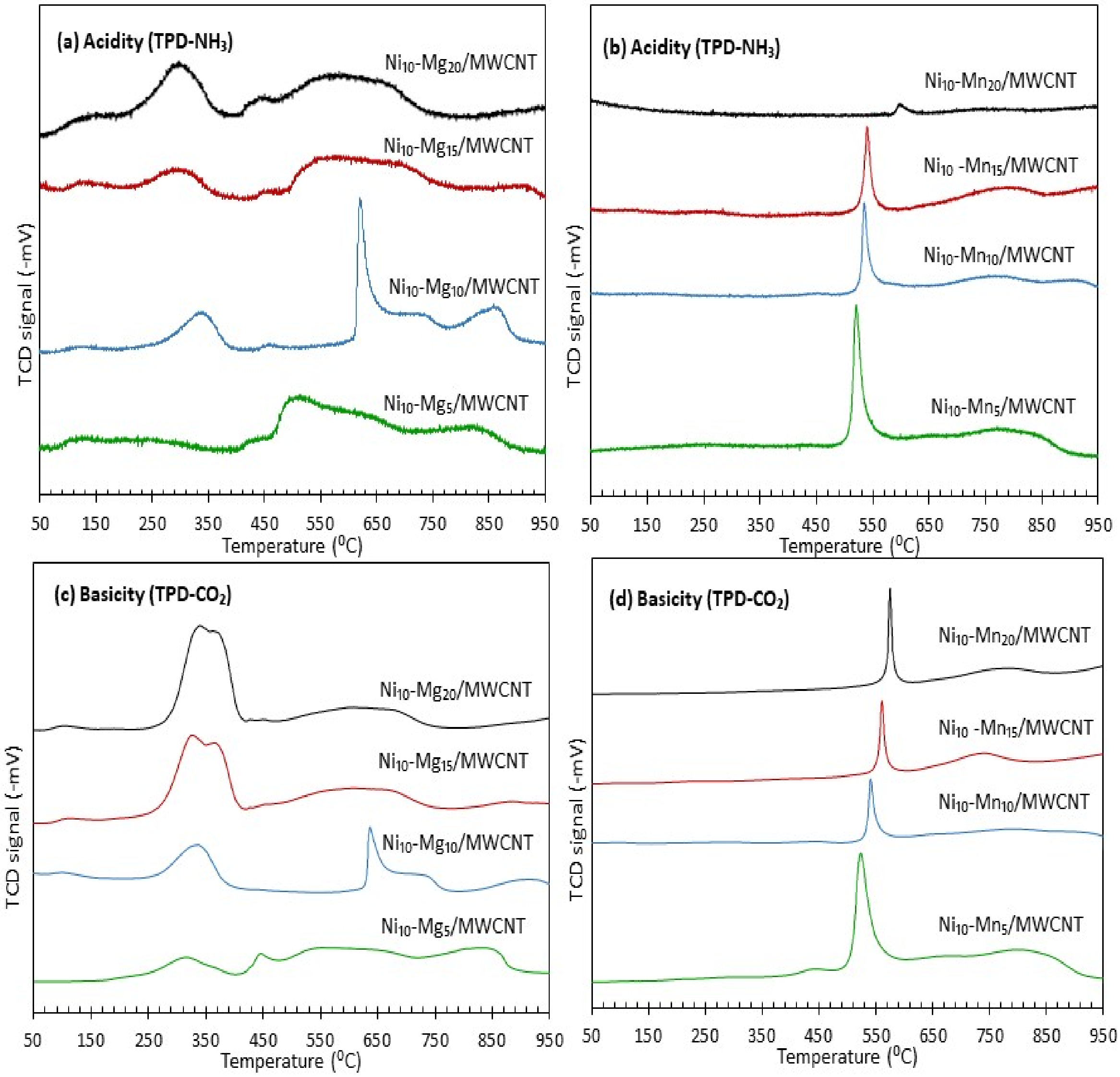

Fig. S4 .TPD-NH_3 analysis of (a) Ni-Mg_n/MWCNT (b) Ni-Mn_n/MWCNT and TPD-CO_2 (c) Ni-Mg_n/MWCNT (d) Ni-Mn_n/MWCNT with different Mg and Mn concentration (n=5-20 wt %).

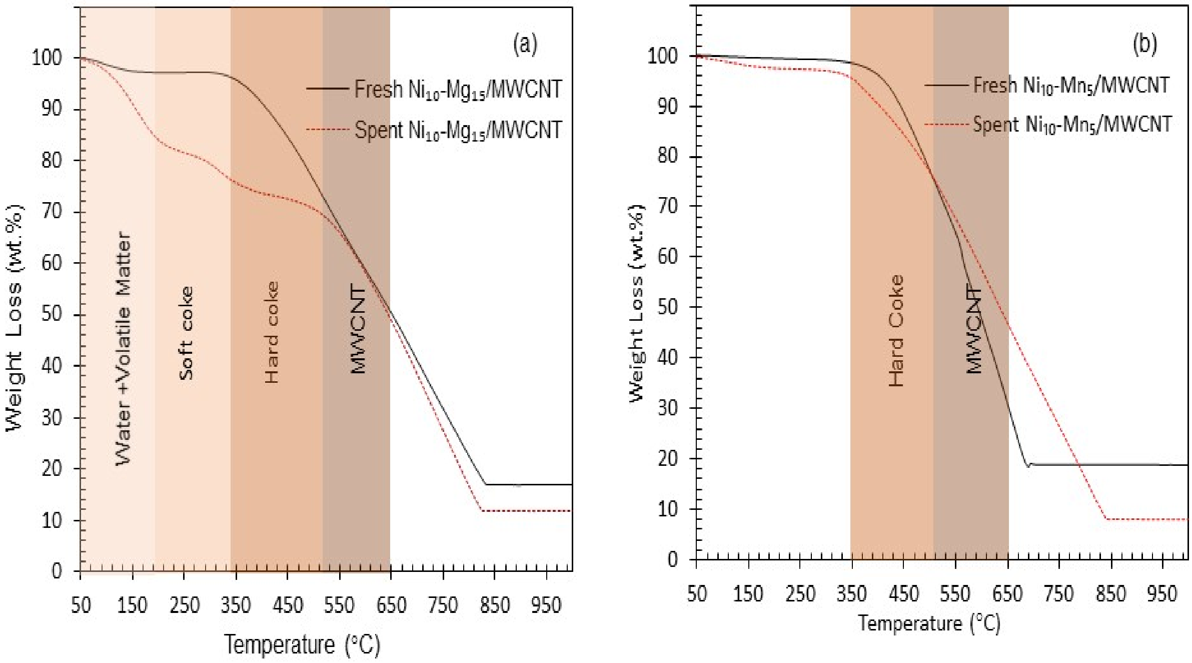

Fig S5. TGA for fresh and spent (a) Ni_{10}-Mg_{15}/MWCNT and (b) Ni_{10}-Mn_5/MWCNT catalysts.
